# Robust acceleration of Earth system heating observed over the past six decades

**DOI:** 10.1038/s41598-023-49353-1

**Published:** 2023-12-27

**Authors:** Audrey Minière, Karina von Schuckmann, Jean-Baptiste Sallée, Linus Vogt

**Affiliations:** 1 Université Toulouse III - Paul Sabatier, Toulouse, France; 2 Mercator Ocean International, Toulouse, France; 3 LOCEAN-IPSL, Laboratoire d’Océanographie et du Climat: Expérimentation et Approches Numériques, CNRS/IRD/MNHN, Sorbonne Université, Paris, France

**Keywords:** Ocean sciences, Physical oceanography, Climate sciences, Climate change

## Abstract

Global heating of the Earth system is unequivocal. However, detecting an acceleration of Earth heating has remained elusive to date, despite suggestive evidence of a potential increase in heating rates. In this study, we demonstrate that since 1960, the warming of the world ocean has accelerated at a relatively consistent pace of 0.15 ± 0.05 (W/m^2^)/decade, while the land, cryosphere, and atmosphere have exhibited an accelerated pace of 0.013 ± 0.003 (W/m^2^)/decade. This has led to a substantial increase in ocean warming, with a magnitude of 0.91 ± 0.80 W/m^2^ between the decades 1960–1970 and 2010–2020, which overlies substantial decadal-scale variability in ocean warming of up to 0.6 W/m^2^. Our findings withstand a wide range of sensitivity analyses and are consistent across different observation-based datasets. The long-term acceleration of Earth warming aligns qualitatively with the rise in CO_2_ concentrations and the decline in aerosol concentration during the same period, but further investigations are necessary to properly attribute these changes.

## Introduction

In the past 150 years, Earth's climate has been warming at a rate that is unprecedented in at least the last 2000 years^1^. This human-caused warming has caused widespread adverse impacts and related losses and damages to nature and people, which will continue in the future as global climate continues to warm^2^. Detecting changes in the rate of warming is crucial for informed decision-making in international climate negotiations, with the aim of limiting global warming to specific levels. However, it remains a significant challenge to detect such changes due to the substantial internal variability of the climate system on a decadal scale (e.g., ref. ^3^). In this paper, we address this challenge by examining the global heat accumulation rate across the entire climate system, including the ocean, atmosphere, cryosphere, and land. By focusing on this integrated view, rather than solely relying on changes in global mean surface temperature, we can mitigate the impact of variability and gain a more comprehensive understanding^4,5^.

**Figure 1:**
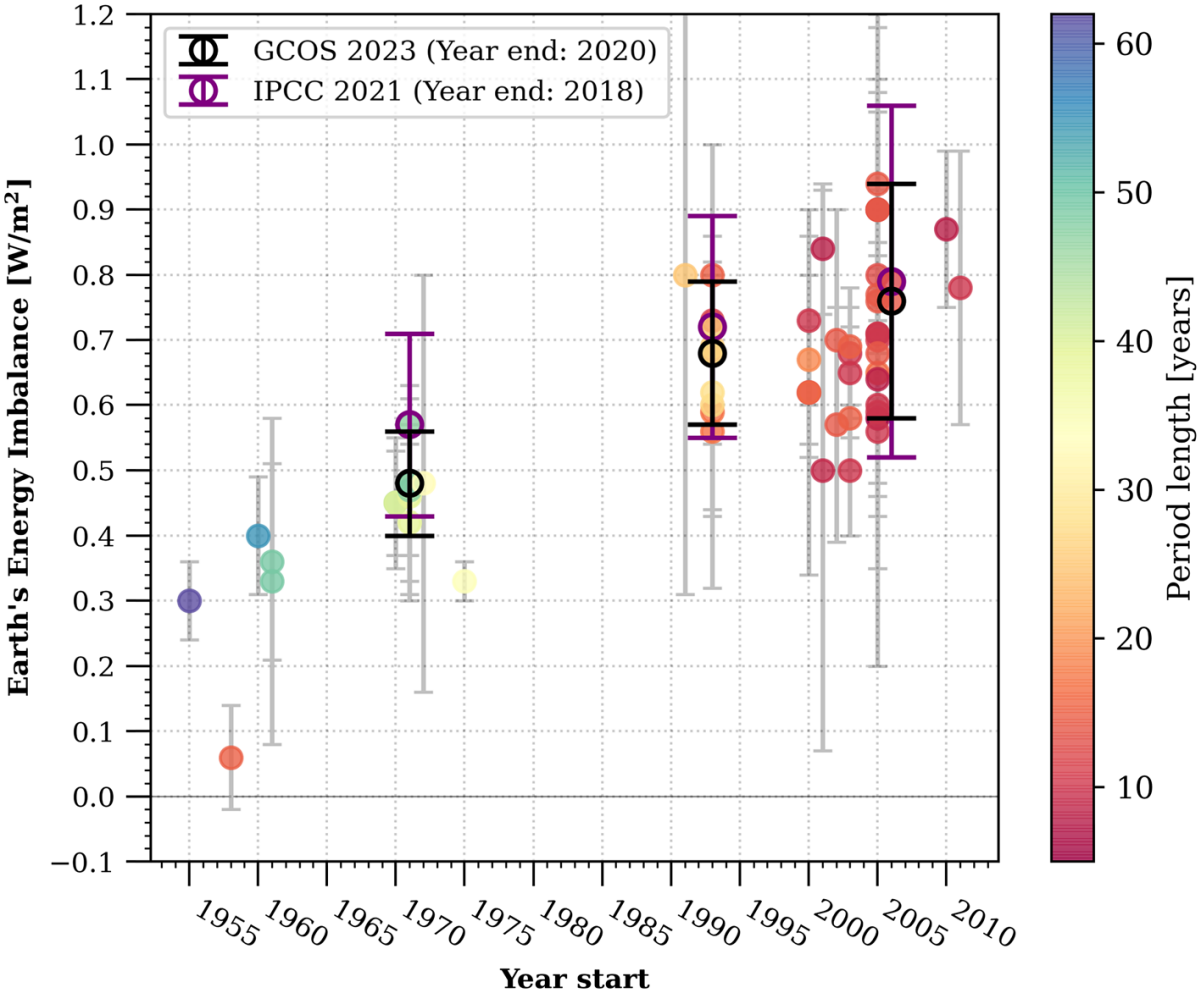
Assessment of observation-based Earth’s energy imbalance (EEI) absolute values as available in the literature, considering various approaches and time periods (refer to Table S1 for references). The black circles highlight the EEI values derived from an international assessment conducted within the Global Climate Observing System^7^ (GCOS) framework. Notably, these values show a significant agreement with the EEI values estimated in the latest IPCC report^4^, which are represented by the purple circles.

Global heat accumulation in the climate system, resulting from the current positive Earth's Energy Imbalance (EEI) at the top of the atmosphere, is primarily dominated by changes in Global Ocean Heat Content (GOHC)^4^. GOHC changes account for approximately 90% of the total heat increase in the past fifty years, while land heating, ice melting, and atmospheric warming contribute around 5%, 3%, and 1% respectively^6–8^. Several studies have indicated an increase in the global heat accumulation rate in recent decades, with values rising from 0.50 [0.32 to 0.69] W/m^2^ during the period 1971–2006 to 0.79 [0.52 to 1.06] W/m^2^ (90% confidence interval) for the period 2006–2018 (ref.^4,6–22^ and Fig. 1). Some studies have even suggested a potential doubling of EEI in the last decade compared to the previous one^6,17^.

Despite this suggestive body of evidence, no study has conducted an analysis of heat accumulation acceleration since 1960 to date. While the results presented in Fig. 1 provide insights, they represent accumulation rates computed over varying time spans, with higher rates calculated over decadal periods and lower rates calculated over multi-decadal periods. This variation in time spans makes it challenging to make definitive and quantified statements about acceleration. Additionally, the use of diverse datasets and methodologies can significantly impact the calculated accumulation rates. The only notable climate variable where acceleration has previously been detected in past decades is Global Mean Sea Level (GMSL)^23–31^. This GMSL acceleration has been attributed to factors such as increasing GOHC leading to thermal expansion of seawater, declining land water storage, or increasing land ice melt^25–27,31,32^.

In this paper, we present the first observation-based quantification of the acceleration of Earth's system heating. Our study adopts a systematic approach, incorporating multiple datasets and employing various methods. We estimate the rate of change and acceleration of Earth's heat content using a collection of GOHC time series derived from in-situ temperature data spanning from 1960 to 2020. Additionally, we utilise ocean reanalyses data from 2005 to 2020 and satellite altimetry and gravimetry data from 2002 to 2020. To complement our analysis, we also incorporate non-ocean heat content time series covering the period from 1960 to 2020, and compare our findings to observations of the net radiative flux at the top of the atmosphere (TOA) spanning from 2001 to 2020.

## Heat content rate of change

Several research groups have developed four-dimensional global ocean temperature datasets, which enable the estimation of GOHC. In this study, we use an ensemble of ten freely accessible products (refer to Table S2 for the exhaustive list and associated references) and take a systematic approach to assess their consistency and discrepancies (see “Methods” section). We compare this ensemble mean to three other estimates of GOHC based on the ensemble mean of three ocean reanalyses, one satellite-derived estimate^20^, and a composite ensemble^7^ of sixteen products developed within the framework of the Global Climate Observing System (referred to as GCOS heat content). It should be noted that not all products cover the same time period (see Fig. S1 and Table S2), and we have aimed to maximise the number of products used for each discussed time period throughout this paper.

None of these GOHC estimates can be considered flawless. The process of producing these estimates is accompanied by significant challenges stemming from observational gaps, historical changes in observational coverage, and potential sensor errors^33–35^. Consequently, each research group must make important assumptions regarding data quality control, data correction, and strategies for filling spatio-temporal gaps. These assumptions collectively contribute to the uncertainty associated with the reconstruction of the GOHC^36–38^. Unfortunately, producers do not always provide the GOHC uncertainty associated with their methodological choices (see Table S2), commonly referred to as internal GOHC uncertainty^39^. Alternatively, one can compute an a posteriori estimate of GOHC uncertainty by determining the structural uncertainty, which is informed by the ensemble spread of a set of products^39–42^. In this study, we aim to investigate how these different estimates of uncertainty, as well as our chosen statistical methodology for inferring time-derivatives, can impact the computation of GOHC rates of change.

**Figure 2:**
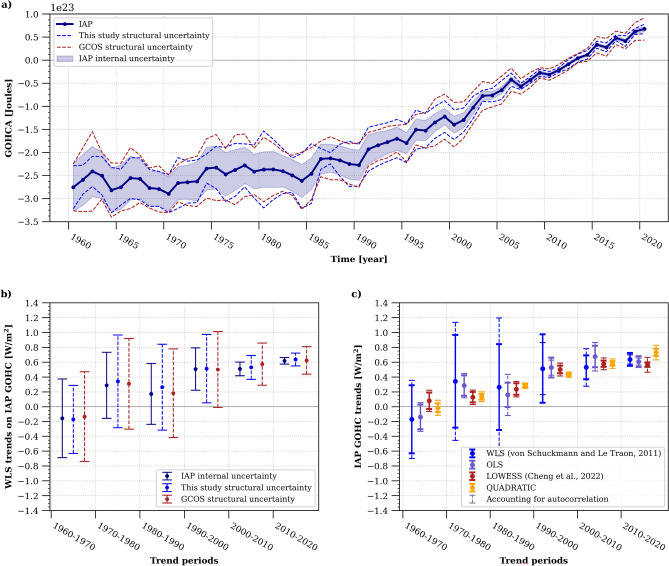
Global ocean heat content (GOHC) rate of change and its corresponding uncertainty estimates. (**a**) Comparison between structural and internal GOHC uncertainties. The GOHC from the IAP product^43^ is represented by the plain blue line, surrounded by its associated internal uncertainty, depicted by blue shading. The dashed blue lines indicate the structural uncertainties derived from this study, while the red dashed lines represent the structural uncertainties from the GCOS product^7^. (**b**) Sensitivity test on GOHC rate of change uncertainties calculations. The bars display two times the WLS regression standard errors using the internal IAP uncertainty (shown in blue) and the structural GOHC uncertainty from this study (indicated by dashed blue), as well as the GCOS product (depicted by dashed red). (**c**) Sensitivity test on GOHC trends computation for different decades. Four methods are evaluated on the GOHC time series for the IAP product: WLS (medium blue), OLS (purple), LOWESS (red), QUADRATIC (yellow). For each method, the trend uncertainties accounting for sample autocorrelation are also shown (dashed lines). Further information on these methods can be found in the Methods section. All uncertainties are shown at the 95% confidence level.

The IAP product^43^ (see Table S2) is one of the few that provides an estimation of its internal uncertainty. We took this opportunity to compare the internal uncertainty estimate of the IAP product with the structural uncertainty of the GCOS heat content^6,7^. Additionally, we calculated our own structural uncertainty based on two times the standard deviation of our set of products (see Methods). Both structural uncertainties encompass the IAP internal uncertainty in the GOHC anomaly time series (Fig. 2a). When propagated to determine the uncertainty of the GOHC rate of change, the structural uncertainty also provides the largest uncertainty estimate (Fig. 2b). Consequently, for the remainder of this study, we will employ the GOHC structural uncertainty in our calculations of GOHC rates of change. We note that our ensemble spread is only indicative of the true structural uncertainty, and might only provide an underestimate of the true uncertainty because we use an ensemble of opportunity. Furthermore, we tested four different methods for computing GOHC rates of change (see “Methods” section). Although the choice of method has minimal impact on the computed rate of change itself, it does influence the associated uncertainty (Fig. 2c). Among the tested methods, the Weighted Least Squares regression (WLS) suggests the largest uncertainty. As a precautionary measure, we have selected to utilise this methodology for the remainder of this paper.

**Figure 3:**
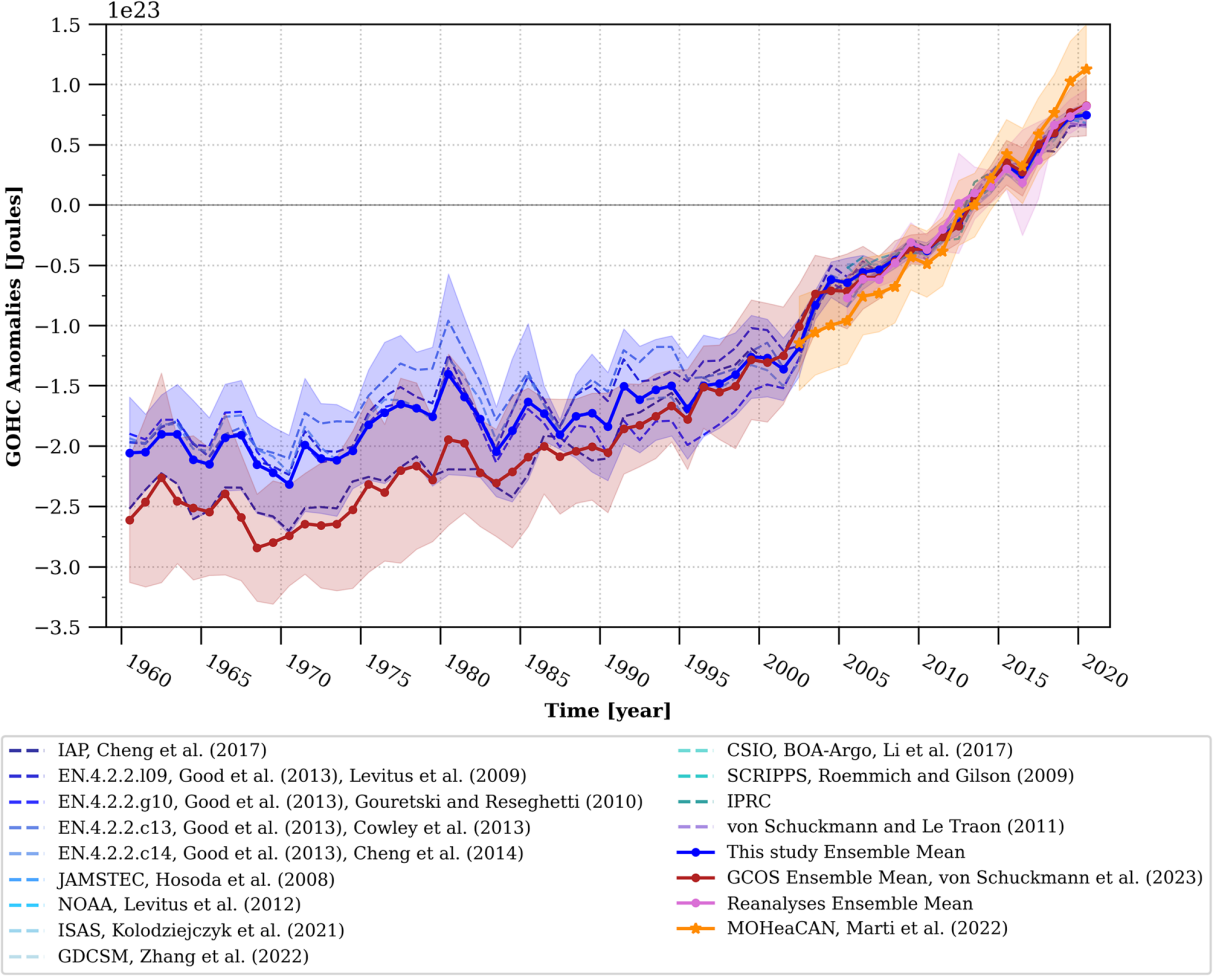
Time evolution of global ocean heat content (GOHC) anomalies. This study's in-situ estimates of GOHC are represented by the blue dashed curve for individual products and the bold blue curve for the ensemble mean. These estimates are compared to the GOHC from GCOS^7^ (red curve), as well as reanalyses (pink curve) and satellite estimates^20^ (MOHeaCAN; orange curve). The shadings on the graph indicate the structural GOHC uncertainties from this study (blue shading), GCOS (red shading), and reanalyses (pink shading). Additionally, the orange shading represents the internal uncertainty corresponding to the satellite-based GOHC estimate^20^. All uncertainties are depicted at the 95% confidence level. The anomalies are presented relative to a baseline period of 2005–2020 (refer to the Methods section for detailed information on the GOHC processing). Refer to Table S2 for product references and additional details.

**Figure 4:**
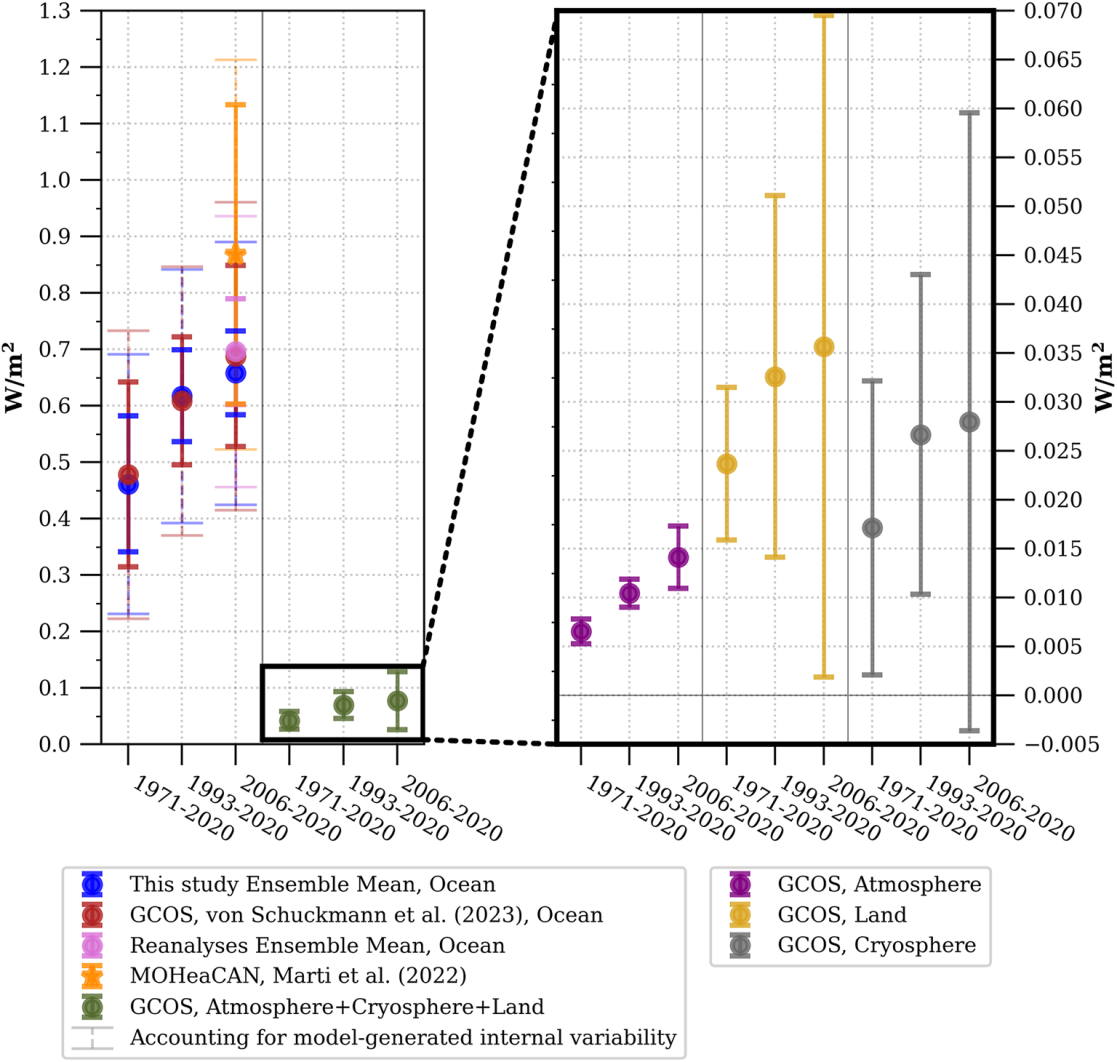
Multi-decadal heating rates for the different components of the Earth System. The heating rates are provided for three main periods of the observing system: historical (1971–2020), satellite (1993–2020) and Argo era (2006–2020). This study’s in-situ estimates of ocean warming rates (blue dots) are compared to GCOS ocean heating rates^7^ (red dots). The non-ocean heating rates (green dots) are computed from GCOS heat content time series^7^, and are the sum of heating rates for atmosphere (purple), land (yellow) and cryosphere (gray). The heating rates and their associated uncertainties are computed using WLS regression and are relative to the Earth's surface at the top-of-atmosphere (as described in the “Methods”). The uncertainties are displayed at the 95% confidence level and take into account serial autocorrelation (see “Methods”) (solid lines). For the ocean heating rates, the uncertainties taking into account model-generated internal variability are also displayed (dashed lines).

Regardless of the methodological choice (Fig. 2), the specific product used, and the time period considered within the past sixty years, all GOHC time series utilised in this study (Fig. 3), along with their associated rates of change (Fig. 4 and Fig. S2), exhibit consistency within their respective uncertainty ranges. This remarkable consistency instils high confidence in the finding that the global ocean has experienced a warming rate of 0.66 ± 0.23 W/m^2^, 0.69 ± 0.27 W/m^2^, or 0.70 ± 0.24 W/m^2^ during the period of 2006–2020, as indicated by the ensemble constructed in this study, the GCOS ensemble, or the ensemble of ocean reanalysis, respectively (Fig. 4) (all uncertainties mentioned here and hereafter are given at the 95% confidence level, and take into account serial autocorrelation and model-generated internal climate variability uncertainty, see Methods, Eq. 12). Only the indirect satellite-derived GOHC estimate suggests a slightly higher rate of change during the period 2006–2020, reaching a value of 0.87 ± 0.35 W/m^2^ (Fig. 4). Nonetheless, all of these warming rates for the period 2006–2020 are greater than rates computed over longer time periods, particularly surpassing the rates for the period of 1993–2020, which stand at 0.61 ± 0.22 W/m^2^ (or 0.61 ± 0.23 W/m^2^ as estimated by GCOS), and significantly exceeding the rates for the period of 1971–2020, which amount to 0.46 ± 0.23 W/m^2^ (or 0.48 ± 0.25 W/m^2^ as estimated by GCOS).

Although the rates of heat content change for the land, atmosphere, and cryosphere are an order of magnitude smaller than the rates for the ocean, all components exhibit higher rates when focusing on more recent decades (Fig. 4). Importantly, this increased warming rate in shorter and more recent time periods is observed to occur at a comparable pace in terms of the percentage of increase across the different Earth system components. Compared to the period of 1971–2020, the in-situ GOHC rate was higher by 34 ± 18% (or 28 ± 24% based on the GCOS estimate) in the period of 1993–2020 and by 42 ± 16% (or 44 ± 34% based on the GCOS estimate) in the period of 2006–2020. The rate of change in heat content for the land component increased by a similar proportion, with a percentage increase of 38 ± 78% and 51 ± 142% for the respective periods. The percentage increase in the rate of change for the cryosphere and atmosphere is also comparable, albeit slightly larger, at 60 ± 22% and 117 ± 49% for the atmosphere, and 56 ± 96% and 64 ± 184% for the cryosphere (see also ref. ^7^).

**Figure 5:**
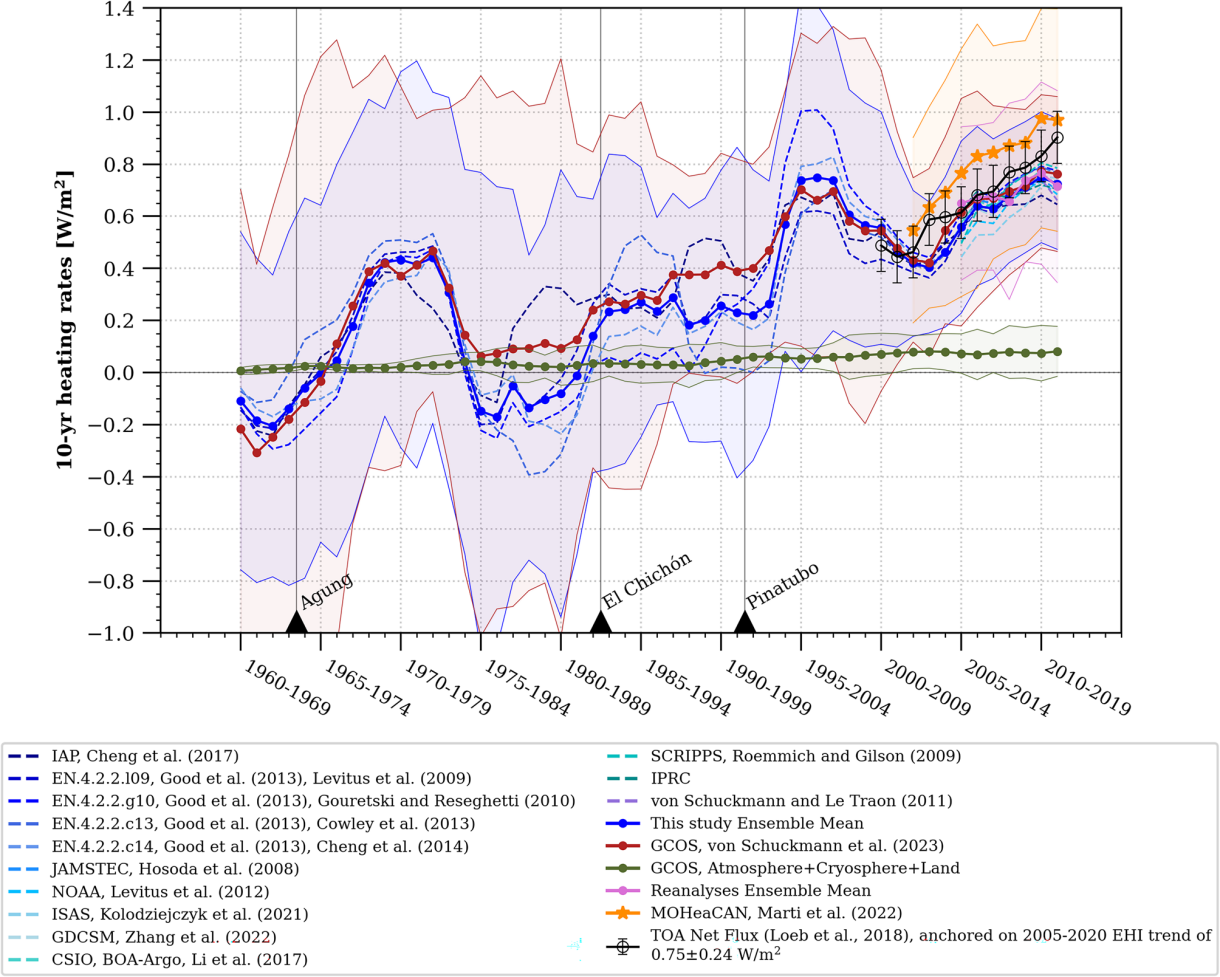
1960–2020 time evolution of decadal heating rates of the Earth. The in-situ estimates of ocean heating rates are represented for each individual product (blue dashed curve, refer to Table S2 for references) and for the ensemble mean (bold blue curve). These estimates are compared to GCOS^7^ (bold red curve), reanalyses (pink curve) and satellite^20^ (MOHeaCAN; orange curve) ocean heating rates. Non-ocean heating rates (green curve) are computed from GCOS heat content time series^7^, and equal to the sum of atmosphere, land and cryosphere heating rates. The 10-year means of the top-of-atmosphere (TOA) net radiative flux (black curve) are anchored on the 2005–2020 Earth Heat Inventory (EHI) trend of 0.75 ± 0.24 W/m^2^ (refer to the Methods section for detailed information on TOA net flux anchoring). Heating rates and associated uncertainties are computed from WLS regression and are relative to the Earth’s surface at the top-of-atmosphere (as described in the “Methods” section). The uncertainties take into account autocorrelation and are shown at the 95% confidence level for all estimates (see “Methods”) except TOA net radiative flux, where uncertainties of ± 0.1 W/m^2^ have been reported^44^. The black triangles indicate major volcanic eruptions that have occurred since 1960. In the context of the heating rates, a positive value indicates that the Earth system is experiencing warming, while a negative value is associated with a cooling.

The increase in the rate of GOHC as we focus on more recent periods aligns with the wide range of estimates from individual published studies and international literature assessments (Fig. 1). However, the precise time evolution of this increase and the impact of comparing periods of different lengths, potentially affected by different processes, remain less clear. To address this, we calculate the heat content rates over consistent 10-year periods using a moving window spanning from 1960 to 2020 (Fig. 5). Despite a large error range in the early years of the time-series and significant decadal variability of up to 0.6 W/m^2^, we observe a clear and steady low-frequency increase in the decadal GOHC rate from 1960 to 2020. The decadal GOHC rate has been consistently rising since the 1960s, with an increase of + 0.91 ± 0.80 W/m^2^ between the first decade (1960–1970) and the last decade (2010–2020) (average across this study and GCOS estimates, Fig. 5).

## Heat content acceleration

**Figure 6:**
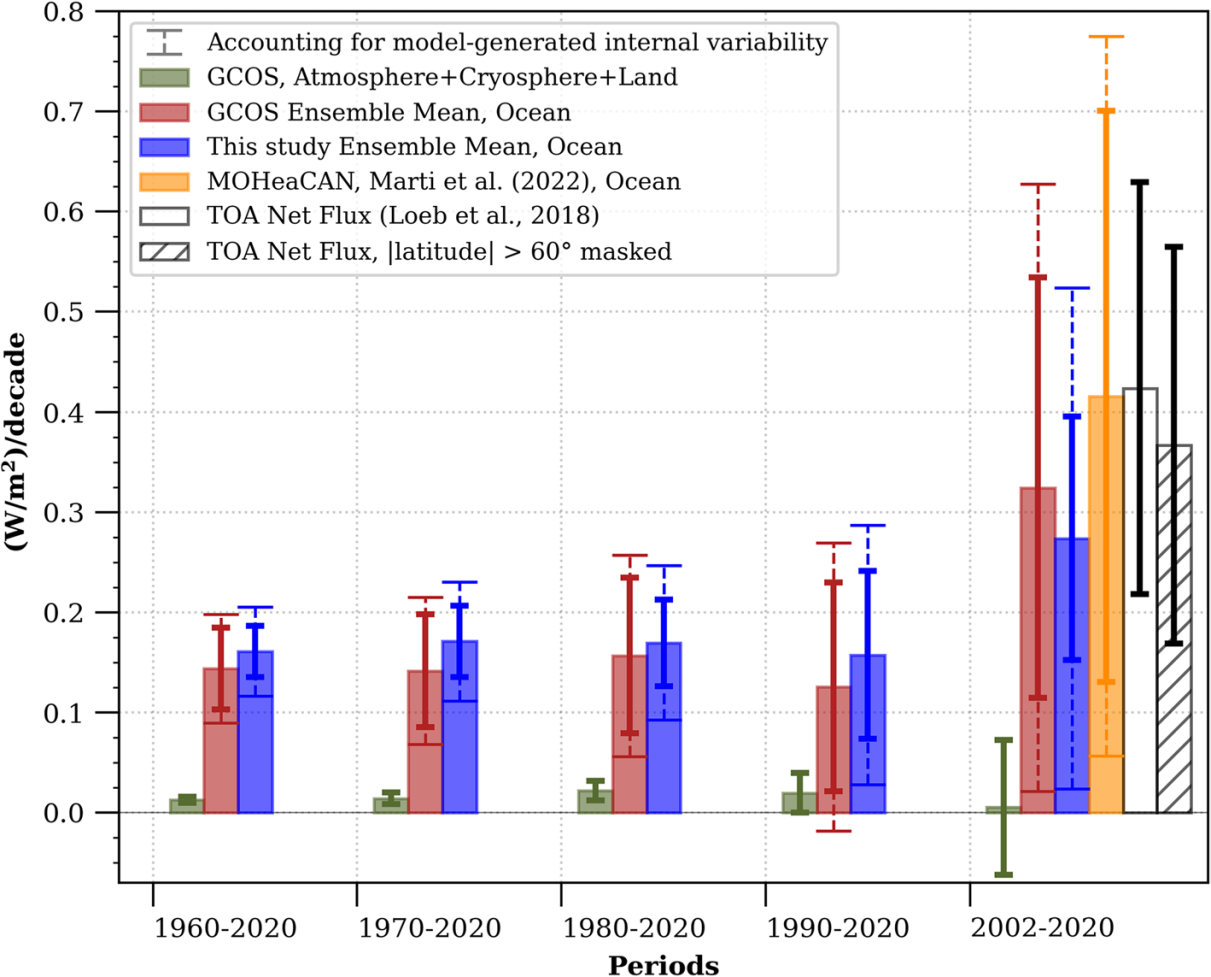
Earth system constant heating acceleration. The acceleration rates are estimated from a quadratic in time WLS regression of the annual GOHC time-series (see “Methods” section). The in-situ ocean estimates from this study (blue bars) are compared to GCOS ocean estimates^7^ (red bars), and satellite ocean estimates^20^ (MOHeaCAN; orange bars). The non-ocean components (green bars) are estimated by summing the atmosphere, land and cryosphere GCOS^7^ heat content time series. The top-of-atmosphere (TOA) estimates of warming acceleration (bars with black contours) are computed from a linear OLS regression accounting for autocorrelation^17,47^, over global TOA net radiative flux (white bar) and near-global TOA net radiative flux (which excludes latitudes higher than 60°, represented by the hatched white bar). Uncertainties for all estimates are shown at the 95% confidence level and take into account serial autocorrelation (solid lines) (see “Methods”). For the ocean heat content accelerations, the uncertainties taking into account model-generated internal variability are also displayed (dashed lines). A positive value indicates that the heat content is accelerating, while a negative value suggests deceleration.

Over the past 20 years, in addition to products based on ocean in-situ measurements, we have gained access to other sources of evidence, such as ocean reanalysis and satellite data, which provide estimates of ocean heat content and energy flux at the top of the atmosphere^44,45^ (Fig. 5). The increase in the Earth's decadal heat content rate estimated from these mostly independent sources of observations is notably consistent and exhibits a clear upward trend (Figs. 4, 5). This leads us to the question: can we formally detect an acceleration of the total Earth's heat content using the existing global climate observing system? To address this question, we employ a WLS methodology to estimate acceleration, consistent with our computation of the rate of change (see “Methods”). We calculate acceleration using a quadratic fit to the annual GOHC estimates shown in Fig. 3. We apply this approach over five multi-decadal periods (1960–2020, 1970–2020, 1980–2020, 1990–2020 and 2002–2020), to the ensemble of GOHC constructed in this study and to the GCOS ensemble, resulting in two acceleration estimates and associated uncertainties for each time period. All of these estimates robustly indicate a significant acceleration of GOHC since 1960, with an average rate of 0.15 ± 0.05 (W/m^2^)/decade. This rate is obtained from the average of the two accelerations and associated uncertainty estimated from this study and the GCOS ensembles while accounting for autocorrelation and the model-generated internal climate variability (see “Methods”, Eq. 12). Importantly, this GOHC acceleration remains remarkably consistent when computed over different multi-decadal time periods (Fig. 6). While not entirely independent, the consistency of the estimated acceleration when using different time periods and observation-based ensembles enhances our confidence in the robustness of this multi-decadal GOHC acceleration. Similarly, acceleration of non-ocean heat content is also significantly detected over these multi-decadal time periods at a rate of 0.013 ± 0.003 (W/m^2^)/decade (green bars in Fig. 6).

The shorter the time period, the more sensitive the quadratic fit is to noise. As a result, the uncertainties associated with the computed acceleration over the past two decades (2002–2020) are much larger compared to those computed over a longer timespan (Fig. 6). Interestingly, however, the computed GOHC accelerations over these two decades remains significant (i.e., the lower bounds of the confidence intervals are above zero), and is notably larger than when computed over a longer timespan. The 2002–2020 in-situ GOHC acceleration is estimated at 0.30 ± 0.28 (W/m^2^)/decade (same as above: average across both this study and GCOS estimates, and accounting for autocorrelation and the model-generated internal climate variability uncertainty based on Eq. 12 in “Methods” section), double the value compared to the 1960–2020 in-situ GOHC acceleration estimate. This substantial in-situ GOHC acceleration estimate in 2002–2020 is supported by two independent estimates: one based on satellite-derived GOHC (0.42 ± 0.36 (W/m^2^)/decade) and the other on satellite-based energy flux at TOA (0.42 ± 0.21 (W/m^2^)/decade). However, our results also indicate that the acceleration estimates over two decades, in contrast to estimates over longer periods, are sensitive to methodological choices. This sensitivity prevents us from drawing firm conclusions regarding increased rate of acceleration over the past two decades (Figs. S4b, c, S5b, c, S6).

## Discussion

Several recent studies have examined changes in GOHC rates over time^16,17,20,46–48^. However, these studies have focused only on the most recent two decades and have not considered changes over a longer period of more than half a century, as we have done in this paper. Using different observational products and methodologies, these studies have estimated an increase in the energy flux at TOA at a rate of 0.42 ± 0.23 (W/m^2^)/decade for the period 2000–2020 (ref. ^47^), or a rate of 0.38 ± 0.24 (W/m^2^)/decade for the period 2001–2020 (ref. ^46^), and an increase in GOHC rates of 0.43 ± 0.40 (W/m^2^)/decade for the period 2005–2019 (ref. ^17^). These previous estimates align well with our multi-product estimate of the acceleration of in-situ GOHC from 2002 to 2020, which is 0.30 ± 0.28 (W/m^2^)/decade. However, it is worth noting that due to the relatively short time span, the acceleration estimate and its associated uncertainty are sensitive to methodological choices. In contrast, our methodology in this study allows us to present compelling evidence that the acceleration of heat content in the Earth system began in the 1960s. GOHC has been steadily accelerating at a rate of approximately 0.15 ± 0.05 (W/m^2^)/decade since then, while other components of the climate system have been accelerating at a rate of 0.013 ± 0.003 (W/m^2^)/decade.

At the multidecadal scale, our findings indicate that the acceleration of Earth's heat content since the 1960s is robust to methodological choices and has remained relatively stable over a span of forty years. This provides empirical evidence supporting the notion that the acceleration is a result of long-term changes in the climate system. The observed multidecadal acceleration in heat content accumulation in the Earth system is qualitatively consistent with the documented likely increase in the rate of total anthropogenic effective radiative forcing since the 1970s, as estimated in the most recent IPCC report^49^. This increase is primarily attributed to the growing concentrations of CO_2_ and the declining concentrations of aerosols^49^.

The rate at which heat accumulates in the Earth system is influenced by three main factors: radiative forcing, physical climate feedback, and land or sea surface temperature, which can modulate the intensity of feedback processes. Radiative forcing encompasses both natural factors (such as solar radiation and volcanic activity) and human-induced factors (such as greenhouse gas and aerosols emissions). The internal variability of the climate system can also affect this heat budget by influencing land or sea surface temperatures^46,50^. The extent to which the observed increase in Earth's heat content rates over the past two decades can be attributed to internal variability or forced by human activities has been a subject of debate^17,46^. The in-situ ocean observation products presented in this study show variability in heat accumulation at a decadal scale, reaching up to 0.6 W/m^2^, but the causality behind these variations remains unclear. The role of internal variability^50,51^, changes in anthropogenic forcing^17,46^, and the presence of uncertainties or undetected biases in the observing system^36,52,53^ in explaining these changes still require further investigation.

In the past two decades, there is some indication that the acceleration of GOHC has increased compared to the previous sixty years, although this finding is sensitive to methodological choices. Raghuraman et al. (2021)^17,46^, using climate model experiments, suggested that it is highly unlikely for the observed 2001–2020 trend in TOA net radiative flux to be solely explained by internal variability. However, they did propose that internal variability could contribute up to ± 0.19 (W/m^2^)/decade over a 20-year period. Therefore, the possible increased acceleration over the past twenty years may result from the combination of internal variability in the climate system superimposed on the lower-frequency acceleration induced by human activities since the 1960s. However, there are also alternative possible explanations that suggest that the acceleration of the past twenty years could be linked to factors such as a significant rise in radiative forcing due to decreased aerosol concentration^48,54–56^, and changes in clouds and sea-ice leading to increased warming due to change in climate feedback^17,57^. Combined, these effects could induce a recent increase in rate of acceleration. To better quantify, understand and attribute this potential recent increase in acceleration, further investigation and quantification are needed.

Although consistent within their uncertainty ranges, it is worth noting a noticeable difference of 0.12 (W/m^2^)/decade between the central estimate of acceleration derived from in-situ ocean observation products (0.30 ± 0.28 (W/m^2^)/decade) and those obtained from remote sensing, such as satellite-derived ocean heat content (0.42 ± 0.36 (W/m^2^)/decade) or satellite-based energy net flux at the top of the atmosphere (0.42 ± 0.21 (W/m^2^)/decade). We should interpret this difference cautiously, considering that it is smaller than the uncertainty associated with each individual estimate. Nonetheless, it does raise questions and emphasises the distinction among the various products used in this study. An important factor contributing to the difference between these products is their spatial coverage. While the satellite-based energy flux estimate encompasses the entire globe, satellite-based ocean heat content excludes latitudes greater than 66°, and ocean in-situ products exclude regions beyond latitudes greater than 60° and the ocean below 2000 m depth. Consequently, we need to consider the potential impact of these different spatial coverages on our results. When we remove high latitudes poleward of 60° from our estimate of energy flux at TOA, the acceleration estimate is reduced by approximately 15%, bringing it closer to the ocean in-situ estimate. However, it is worth noting that satellite-based ocean heat content, which also excludes polar regions, still produces an acceleration estimate consistent with the energy flux at TOA. Therefore, a more plausible factor contributing to the difference may lie in the contribution of the deep ocean below 2000 m. Due to the lack of in-situ ocean coverage below 2000 m, we are unable to quantify the acceleration in this part of the ocean globally. However, one study has reported acceleration of deep ocean warming below 2000 m in the South Pacific Ocean^58^. Additionally, Bagnell and DeVries (2021)^18^ attempted to reconstruct global deep ocean temperature change over the past century and demonstrated a significant increase in the rate of deep ocean warming from the 1990s-2000s, following a cooling phase that may have delayed the acceleration of full-depth GOHC.

Our findings are based on a comprehensive set of products derived from complex datasets that have inherent limitations in their coverage of vast ocean areas. Dealing with errors and uncertainties presents a significant challenge, especially when detecting trends and acceleration^3^. In this study, we have addressed these challenges by testing our results using various approaches to represent and propagate uncertainties in trends and acceleration (see Supplementary Information). Furthermore, we have included a diverse range of products, each employing different methodologies to construct their datasets. Through extensive sensitivity analyses, our results have consistently shown robustness, thereby increasing confidence in their validity. However, it is important to acknowledge that uncertainties persist, particularly concerning the limitations of the observing system during the early years of the analysed period^36,59^.

We reveal a previously uncharted acceleration of the GOHC since the 1960s. These observations serve as crucial indicators of climate change and play a vital role in enhancing our understanding of the Earth's response to human activities. In addition to quantifying the acceleration, our findings highlight the consistent insights provided by the current global climate observing system into past changes in Earth's heat content. It is imperative to prioritise the maintenance and improvement of the global climate observing system to ensure its continued effectiveness in monitoring climate change in the future^7^. Furthermore, expanding the coverage of the global climate observing system to currently undersampled ocean regions and addressing data gaps in non-ocean components^6,7^ would enable more refined analyses of acceleration in ocean warming and reduce uncertainties in detecting and attributing global climate change.

## Methods

### In-situ GOHC timeseries and structural uncertainty

The global ocean heat content (GOHC) constitutes the major pillar (~ 90%) of the Earth Heat Inventory^6,7^, and currently, its rates of change provide the most accurate estimate of the absolute value of the Earth’s energy imbalance.

When the four-dimensional gridded temperature datasets were available (see Table S2), we computed the GOHC (in Joules) at each month *t* by integrating the temperature T between 0 and 2000 m over the global ocean surface, as follows:

1 $$GOHC(t) =\rho *C* {\sum }_{x}{\sum }_{y}{\sum }_{z}T(t, x,y,z)*h(x,y,z)*A(x,y),$$

where h is the layer thickness in metres, ρ = 1030 kg/m^3^, the reference water density, C = 3980 J/°C/kg, the heat capacity of water, and A, the grid cell area at longitude and latitude (x,y) in m^2^. We used a common mask for all gridded products, i.e., the most restrictive mask between all products (here the IPRC product is the one which has the smaller ocean domain), after masking the polar regions (i.e., poleward of 60° latitude) and shallow ocean areas (i.e., where bathymetry is less than 300 m). To account for deep ocean contribution (i.e., ocean below 2000 m), we added a linear trend on GOHC time series of 0.97 ± 0.48 ZJ/year (0.06 ± 03 W/m^2^) from 1992 to 2020 (ref. ^6,7^). We then computed annual averages of GOHC and calculated the GOHC anomalies relative to the 2005–2020 mean. The structural uncertainty is then computed by taking twice the ensemble standard deviation of GOHC anomalies. Note that in cases where structural uncertainty displays long-term variance (e.g., surpassing a 15-year period), the act of baselining each product over a 16-year period (2005–2020) may artificially reduce the inter-product spread over the baseline period.

### TOA net flux anchoring

The net radiative flux at the top of the atmosphere provides one of the most accurate estimates of the time evolution of the Earth's Energy Imbalance to-date, which can be determined to within 0.3 (W/m^2^)/decade (ref. ^60^). However, its absolute value is more uncertain. For example, uncertainty resulting from calibration alone is 2 W/m^2^ (ref. ^61^). There are also other sources of uncertainties associated with radiance-to-flux conversion and time interpolation (~ 0.2 W/m^2^ for each)^60–62^, or in assuming a 20 km reference level (0.1 W/m^2^)^63^. Currently, the net imbalance from the standard CERES data products is ~ 4.3 W/m^2^ (ref. ^44^) which is much larger than the expected EEI to be 0.5-1W/m^2^ (ref. ^5^). Therefore, to overcome this issue in its absolute value, the TOA net flux is commonly adjusted to be consistent with an estimate from ocean in-situ temperature change^12,17^. We chose to offset the TOA net radiative flux time-series to match the Earth Heat Inventory rate of change over 2005–2020 estimated from the GCOS ensemble at 0.75 ± 0.24 W/m^2^ (ref. ^7^), such that TOA net radiative flux mean value over the 2005–2020 period is equal with the GCOS trend value. Applying this offset allows us to plot the TOA net radiative flux time-series on the same axis as other estimates, and has no implication on the calculation of TOA net flux trends.

### Heat content trend evaluation

There are many ways of estimating trends in a time series in the field of climate research (see for example the ref. ^64^). We focus here on the most classical and often used techniques for estimating trends in the field of GOHC research. In Fig. 2, we tested the sensitivity of GOHC rates of change and its uncertainties to four methods which can be grouped into two types of calculations. One is based on a delta approach^39^, and another one is based on a linear least squares approach (e.g., ref. ^37,65–68^). For the uncertainty on the trend analysis, we have to consider four different aspects, i.e., statistical uncertainty (computed from the variance of the fit residuals), data uncertainty (structural or internal), uncertainty for serial autocorrelation, and uncertainty from internal climate variability. Both delta and least square approaches are applied on annual heat content timeseries and are described below, together with relevant uncertainty considerations.

### Delta approach

For the delta approach, the change in heat content series y(t) over a specific period, Δy, is calculated by subtracting the first value from the last value over a specific period. We then computed the linear trend, y_t,_ over the same period by dividing the change (in Joules) by the length of the period (in seconds). This method is widely used in the literature for estimating GOHC linear trends (e.g., ref.^4,11,69^). To reduce the effect of high-frequency variability, data noise or changes in the observing system, before computing the trend, we first smoothed the time series y(t), using a weighted scatterplot smoothing approach^7,59^ (named LOWESS in Fig. 2) , or a quadratic fit (named QUADRATIC in Fig. 2). For the LOWESS fit, we use a span width of 25 years following Cheng et al. (2022) (ref. ^7,59^).

To obtain an uncertainty range on our estimate of the rate of change, and to take into account the sensitivity of the calculation to interannual variability, we implemented a Monte-Carlo bootstrap to generate 1000 surrogates’ series, y_random_(t), under the assumption of a given mean (our fitted time series, y_fit_(t))^7,59^. Each surrogate y_random_(t) consists of the fitted time series y_fit_(t) plus a randomly generated residual which follows a normal (Gaussian) distribution of standard deviation equal to the uncertainty associated to the time series y(t). We have then repeated this approach, now randomly generating surrogates, y_random_autocorr_(t), following a Gaussian distribution with a standard deviation equal to the residual variance, and including an autocorrelated component structured according to an AR(1) process (see Fig. 2b of ref. ^59^). For both cases, the surrogates are smoothed with a LOWESS or QUADRATIC fit, and the trends are estimated from it, noted respectively y_random, t_ and y_random_autocorr, t_. To associate an uncertainty to the trend, y_t_, we compute the standard deviation of all the 1,000 trends y_random, t_, noted as σ_random_, and the standard deviation of all the 1,000 trends y_random_autocorr, t_, noted as σ_random_autocorr_. The 95% confidence interval for the linear trend, y_t,_ is then calculated as follows:

2 $$CI = \pm 2*\sqrt{{\sigma }_{random}^{2}+{\sigma }_{random\_autocorr}^{2}}.$$

With this method, we consider the data uncertainty, and we account for serial autocorrelation.

### Least squares approach

The Ordinary Least Square (OLS) approach is a classical method for estimating trends in key climate variables such as global mean surface temperature (GMST), global mean sea level (GMSL) or GOHC (e.g., ref.^8,37^). The standard error of OLS regression can be adjusted to consider the serial autocorrelation that can be very strong in the time series of climate variables, such as GMST^70–72^ or radiative fluxes at the top of the atmosphere^17,47^. However, the OLS regression does not consider the uncertainty associated with the variable for which we aim to estimate the trend, which is why some studies use other methods such as the Weighted Least Square (WLS) regression (e.g., ref.^37,66–68^). In this study, we consider these types of regressions together, with the aim of choosing the most suitable method for our case study in terms of estimating uncertainties and trends.

We regressed the equation y = βt + ε, where y is the observed quantity (here the GOHC series), t is the time vector, β are unknown regression coefficients and ε are the associated errors which are assumed to be Gaussian with mean zero. The regression was performed using either an ordinary least squares (named OLS in Fig. 2), or a weighted least squares (named WLS in Fig. 2) regressions.

The OLS regression estimates β_OLS_ and their associated variances are given by the following equations (ref. ^65^):

3 $${\widehat{\beta }}_{OLS} = {{(X}^{T}X)}^{-1}{X}^{T}Y,$$

and:

4 $${var(\widehat{\beta }}_{OLS}) = {\widehat{\sigma }}^{2}{{(X}^{T}X)}^{-1},$$

where X is the design matrix (with ones in the first column and time values in the second column), Y = y^*T*^*.* In Eq. (4), $\widehat{\sigma }$ is the standard error of the regression, computed as:

5 $${\widehat{\sigma }}^{2}=\frac{1}{N-2}\times {\sum }_{i=1}^{N}{{e}_{i}}^{2},$$

where N is the sample size, and *e* are the residuals of the regression.

To take into account the data uncertainty in the regression, we use a WLS fit, which estimates β_WLS_ and their associated variances, as follows (ref.^65,67^):

6 $${\widehat{\beta }}_{WLS} = {{(X{^{\prime}}}^{T}X\mathrm{^{\prime}})}^{-1}{X{^{\prime}}}^{T}Y{^{\prime}},$$

and:

7 $$Var(\widehat{\beta }{ }_{WLS})={{(X}^{T}WX)}^{-1},$$

with:

8 $$W= diag(\frac{1}{{{w}_{ii}}^{2}}),$$

where W is a weighting matrix in which w_ii_ are chosen to be the uncertainties associated to *y* (for example the structural uncertainty of in-situ GOHC time series), $Y{\prime}={W}^\frac{1}{2}Y$ and $X{\prime}={W}^\frac{1}{2}X$. The advantage of WLS over OLS lies in its capacity to account for data uncertainty in the regression parameters and their associated standard errors. Note that for GOHC trend and acceleration evaluation, we only take into account the structural/internal uncertainty in the weighted matrix, and we do not consider the uncertainty raising for internal climate variability (denoted by σ_ICV_). Assuming that σ_ICV_ is constant over time: if σ_ICV_ significantly surpasses the structural/internal uncertainty, the w_ii_ values would remain constant, and trend (or acceleration) values would closely resemble those from an OLS regression. Our evaluation shows that both OLS and WLS methods yield similar results within the ranges of uncertainty (Fig. S4 and S5). We adopt a conservative approach by preferring WLS, which is associated with uncertainties larger than OLS.

Both OLS and WLS methods assume white noise for fit residuals, i.e. they suppose no serial autocorrelation. However, we know that annual ocean heat content series exhibit autocorrelation (ref. ^59^). We show that the residual autocorrelation structure of the heat content series is well captured—or even overestimated—by an autoregressive AR(1) process (see Supplementary Information Fig. S7). We then follow the methodology of Santer et al. (2008) (ref. ^71^) to compute a correction factor in order to adjust the standard error of the OLS and WLS parameters (computed in Eqs. 4 and 7 respectively). This correction factor is obtained via:

9 $$\upsilon = \frac{1 + \rho }{1 - \rho },$$

with $\rho$ the lag-1 (for 1-year) temporal autocorrelation coefficient of the regression residuals. Additionally, we test the influence of significant volcanic eruptions on the autocorrelation pattern of residuals. We show that the lag-1 coefficient for both the GCOS dataset and the dataset in our study exhibits minimal changes when discarding years after significant volcanic eruptions (as depicted in Fig. S7 of the Supplementary Information). As a result, we decide to retain the entire GOHC time series without excluding years following major volcanic eruptions. The corrected standard error, σc, is then computed as:

10 $${\sigma }_{c}={\sigma }\sqrt{\upsilon },$$

To compute the TOA net radiative flux trends, we use an OLS fit and adjust the standard error based on Eq. (9), as in Loeb et al. (2021) (ref. ^17^).

The 95% confidence interval for the trend is calculated based on ± 2 times the corrected standard error of the regression as following:

11 $$CI = \pm 2{\sigma }_{c},$$

### Heat content acceleration evaluation

To compute heat content accelerations shown in Fig. 6, we regress a quadratic fit from the yearly heat content time series using a second-order WLS regression (i.e. we add a third column including a quadratic term *t*^*2*^ in the design matrix X of Eqs. 5 and 6), using the GOHC structural uncertainty as weighting matrix. As for the linear least squares approach, the 95% confidence interval for the acceleration is calculated based on ± 2 times the corrected standard error of the regression (Eq. 10). We also discuss another approach to detect acceleration on Supplementary Information (see Discussion S3 and Figs. S4, S5, S6, S10).

### Sensitivity of GOHC trend and acceleration estimates to internal variability

To assess the contribution of internal variability to trend values (Figs. 4 and 5) and accelerations (Fig. 6), we use pre-industrial simulations of GOHC from an ensemble of climate models of the CMIP6 exercise (46 models, each with one realisation), following the methodology outlined by Raghuraman et al. (2021, 2023) (refs. ^46,73^). For each of the X-length periods of interest, we randomly selected 3,000 segments of GOHC of length X from the model ensemble. From these segments, we calculated trends and accelerations using linear and quadratic fits (see Figs. S8 and S9 in the Supplementary Information). Subsequently, we computed the standard deviation of the 3000 trend and acceleration values, denoted as σ_ICV_ (see Figs. S8 and S9 in the Supplementary Information). We use σ_ICV_ as a proxy to quantify the portion of uncertainty in trends and acceleration introduced by internal variability, which we incorporate into confidence intervals as follows:

12 $$C{I}_{ICV} = 2 * \sqrt{{\sigma }_{c}^{2}+{\sigma }_{ICV}^{2}},$$

where ${\sigma }_{c}$ represents the adjusted standard error of the linear and quadratic fit as described in Eq. (11). This new confidence interval is depicted by the dashed error bars in Figs. 4, 5, and 6.

### Reference surface

To ensure consistency with TOA net radiative flux estimate, all the heat content trend and acceleration values are given relative to the Earth’s surface at the top-of-atmosphere, S_TOA_, computed as follows: S_TOA_ = 4π(R_T_ + z_TOA_)^2^ with R_T_ the Earth’s radius equals 6371 km, and z_TOA_ the altitude of the top-of-atmosphere equals 20 km^20,63^.

### Supplementary Information

Supplementary Information.

## Data Availability

All datasets used in this study are freely available and can be downloaded from websites listed in Table S2.
