# Supplementary material for: Robust acceleration of Earth system heating observed over the past six decades
Source: Sci Rep. 2023 Dec 27;13:22975. doi: 10.1038/s41598-023-49353-1 (PMC10752897; doi:10.1038/s41598-023-49353-1)
Supplement: Supplementary file 1 — Supplementary Information. [file 41598_2023_49353_MOESM1_ESM.pdf]

Supplementary Informations for

Robust acceleration of Earth system heating observed over the past six decades

Audrey Minière[1,2], Karina von Schuckmann[2], Jean-Baptiste Sallée[3] & Linus Vogt[3]

[1] Université Toulouse III, Paul Sabatier, Toulouse, France

[2] Mercator Ocean International, Toulouse, France

[3] Sorbonne Université, CNRS, LOCEAN, Paris, France.

Corresponding author: Audrey Minière (aminiere@mercator-ocean.fr)

Supplementary Figures

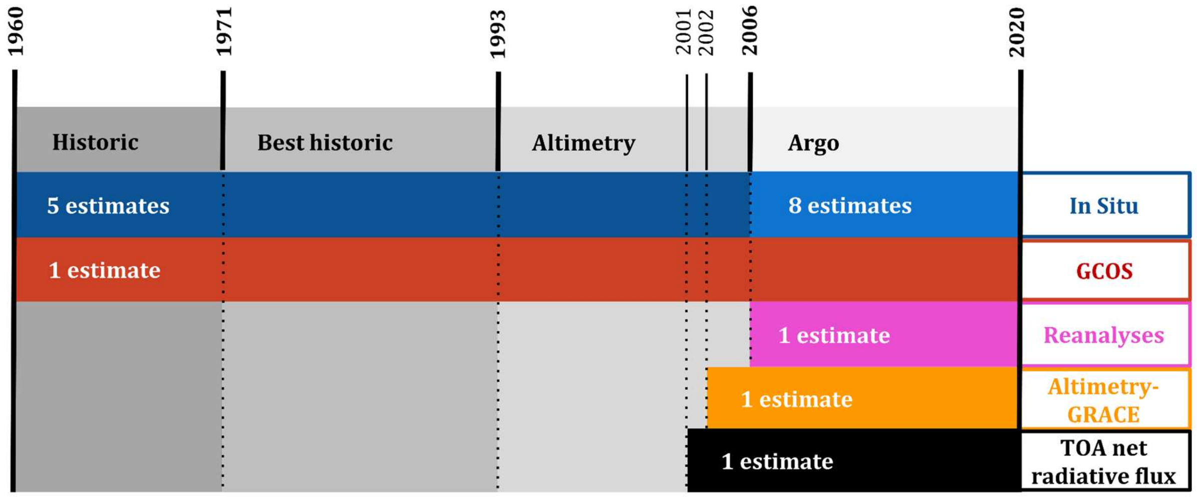

Figure S1 | Schematic illustration showing the time coverage of products used in this study and the main eras of Global Climate Observing System (GCOS), i.e., historic era (1960-2020), best historic era (1971-2020, i.e., as defined in IPCC reports), altimetry era (1993-2020) and Argo era (2006-2020).

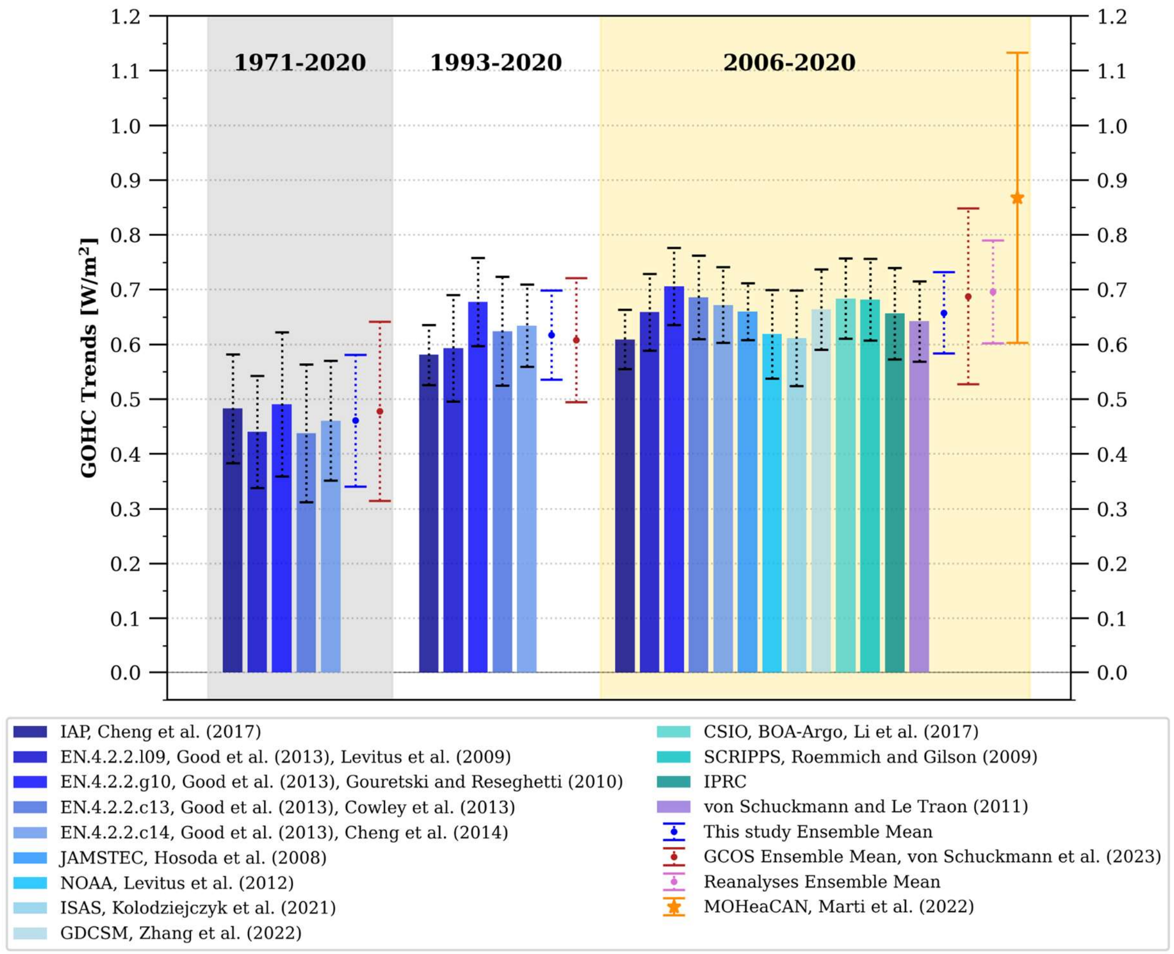

Figure S2 | Global ocean heat content trends per product over the best historic era (1971-2020), altimetry era (1993-2020) and Argo era (2006-2020), as defined in Fig. S1 and in IPCC reports. All the trends are calculated using a WLS regression (see Methods). The trends for each individual product (coloured bars) were computed using this study GOHC structural uncertainty in the weighting matrix (see Methods). The trends for ensemble mean based estimates (i.e., this study in blue, GCOS in red, reanalyses in pink) were computed using their associated GOHC structural uncertainty in the weighting matrix. The trend for satellite-based product (orange dot) is obtained using its associated GOHC internal uncertainty in the weighting matrix. The error bars highlight two times the autocorrelation-adjusted standard error of the WLS regression based on GOHC structural uncertainty (dashed line) or on the GOHC internal uncertainty (solid lines). See Table S2 for product references and additional details. We note that all in-situ products agree in estimating trends across time periods and that the estimate based on this study GOHC ensemble mean is remarkably consistent with the estimate based on the GCOS time series. The estimate based on the reanalysis product also shows a very similar trend to the in-situ

estimates over 2006-2020. The satellite-based product shows a higher trend than the in-situ and reanalysis estimates over the period 2006-2020, but is consistent in terms of uncertainty intervals.

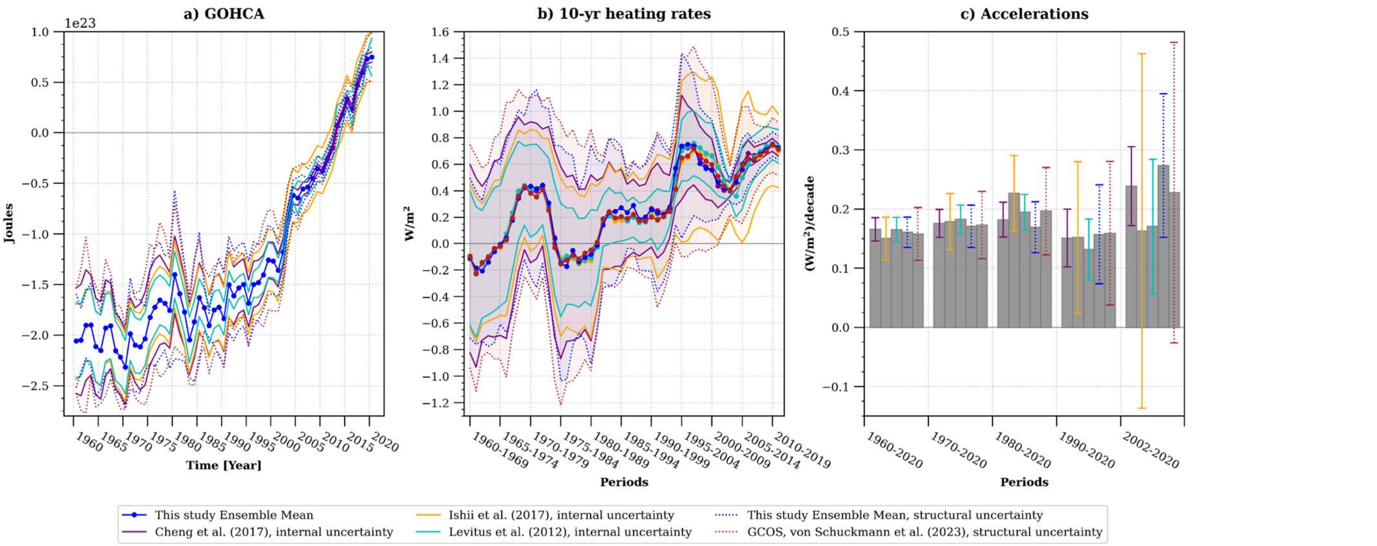

Figure S3 | Influence of uncertainty used in the calculations of GOHC trends and accelerations. **(a)** GOHC ensemble mean time series of this study (dark blue) and its structural uncertainty (dashed blue), the GCOS structural uncertainty (dashed red), the IAP internal uncertainty[3] (purple), the Levitus internal uncertainty[34] (cyan) and the Ishii internal uncertainty[44] (yellow). **(b)** Decadal GOHC trends computed from a WLS regression using this study (blue), GCOS[9] (red), IAP[3] (purple), Levitus[34] (cyan) and Ishii[44] (yellow) uncertainties in the weighting matrix (see Methods). **(c)** GOHC accelerations using quadratic WLS regression with this study (blue errorbar), GCOS[9] (red errorbar), IAP[3] (purple errorbar), Levitus[34] (cyan errorbar) and Ishii[44] (yellow errorbar) uncertainties in the weighting matrix (see Methods). All uncertainties are shown at the 95% confidence level and consider serial autocorrelation.

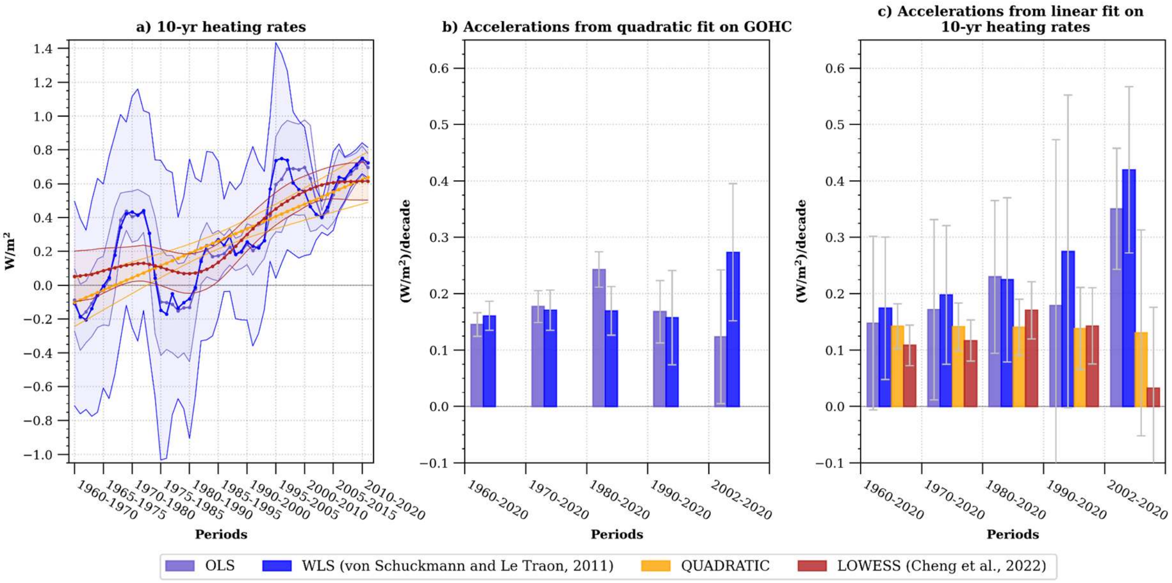

Figure S4 | Sensitivity of GOHC ensemble from this study to the method used to estimate trends and accelerations. (a) Decadal GOHC trends computed from an OLS regression (purple), a WLS regression (dark blue), a LOWESS fit (red) and a QUADRATIC fit (yellow). GOHC accelerations using **(b)** a quadratic regression on GOHC timeseries and **(c)** a linear fit over 10-year heating rates over different multidecadal periods. Note that the LOWESS and QUADRATIC methods do not allow for quadratic regression because they are based on a Delta approach (see Methods). For the WLS, LOWESS and QUADRATIC methods, we use this study GOHC structural uncertainty (the OLS method does not use the GOHC uncertainty in the calculation, see Methods). All uncertainties are shown at the 95% confidence level and consider serial autocorrelation.

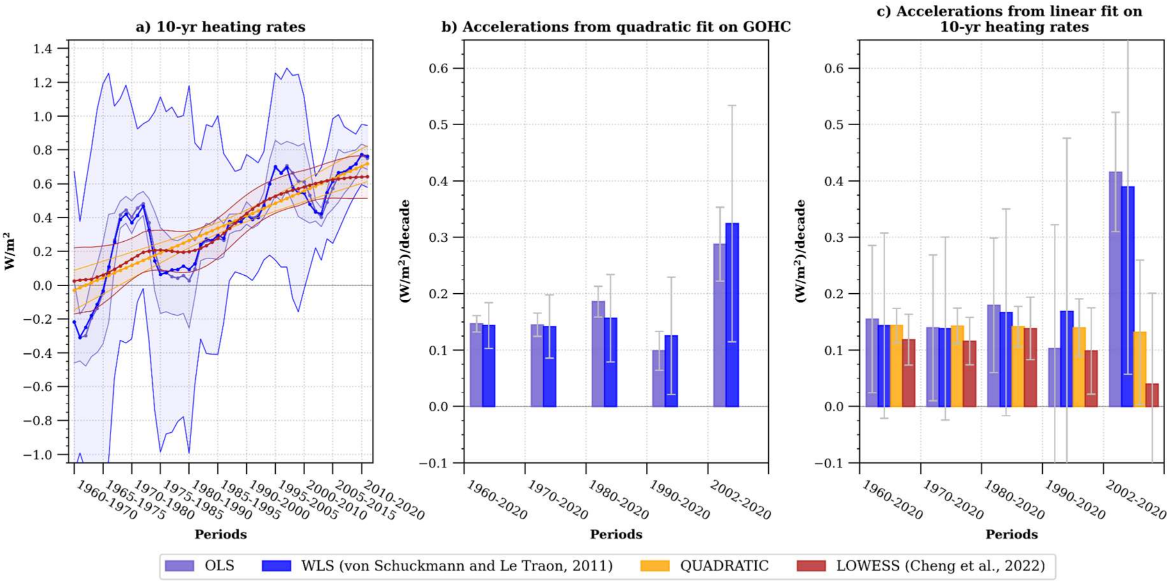

Figure S5 | Sensitivity of GOHC ensemble from GCOS to the method used to estimate trends and accelerations. (a) Decadal GOHC trends computed from an OLS regression (purple), a WLS regression (dark blue), a LOWESS fit (red) and a QUADRATIC fit (yellow). GOHC accelerations using **(b)** a quadratic regression on GOHC timeseries and **(c)** a linear fit over 10-yr heating rates over different multidecadal periods. Note that the LOWESS and QUADRATIC methods do not allow for quadratic regression because they are based on a Delta approach (see Methods). For the WLS, LOWESS and QUADRATIC methods, we use GOHC structural uncertainty from GCOS (the OLS method does not use the GOHC uncertainty in the calculation, see Methods). All uncertainties are shown at the 95% confidence level and consider serial autocorrelation.

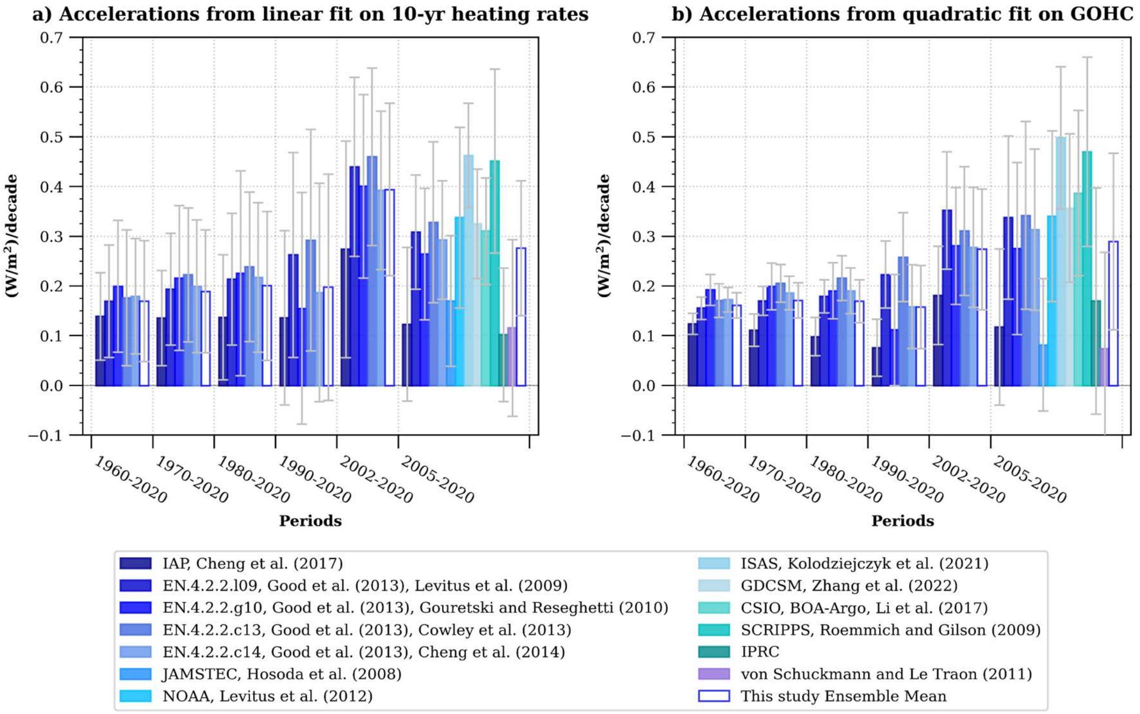

Figure S6 | Sensitivity to the product used to estimate the GOHC acceleration. GOHC accelerations using **(a)** a WLS linear regression over 10-year GOHC heating rates and **(b)** one quadratic WLS regression over GOHC timeseries and over different multidecadal periods. The acceleration values are estimated for each product used in this study GOHC ensemble (solid bar) and for the ensemble mean (blank bar). For the WLS regression of each individual product, we use the GOHC structural uncertainty of this study in the weighting matrix, as for the WLS regression of this study GOHC ensemble mean. All uncertainties are shown at the 95% confidence level and consider serial autocorrelation.

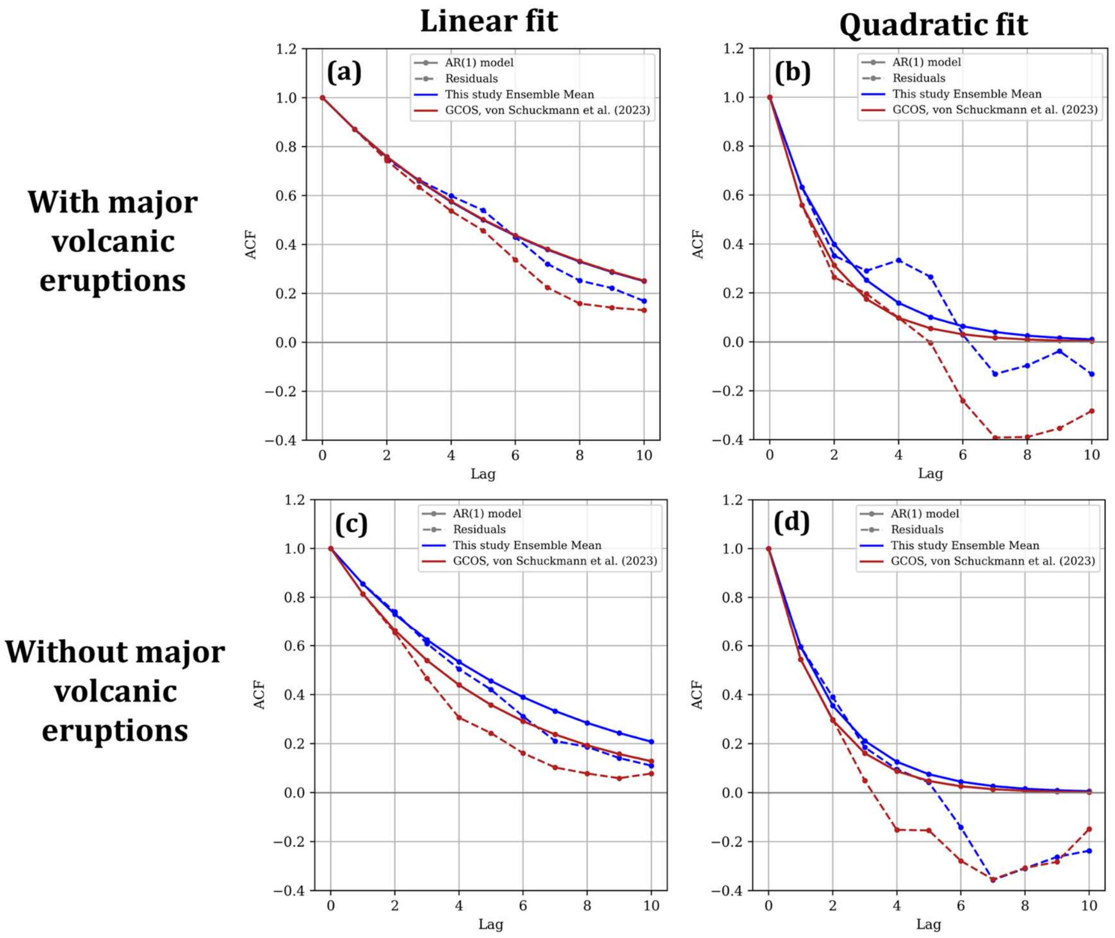

Figure S7 | Analysis of autocorrelation structure of fit residuals. Autocorrelation function (ACF) of residuals from **(a)** linear and **(b)** quadratic fits to GOHC time series within 1960–2020 based on GCOS (dashed red) and this study (solid blue) datasets, and compared to the estimated AR(1) model (solid lines). Same as (a) and (b) for **(c)** and **(d)** respectively, but after excluding the 3 years following a volcanic eruption in GOHC time series.

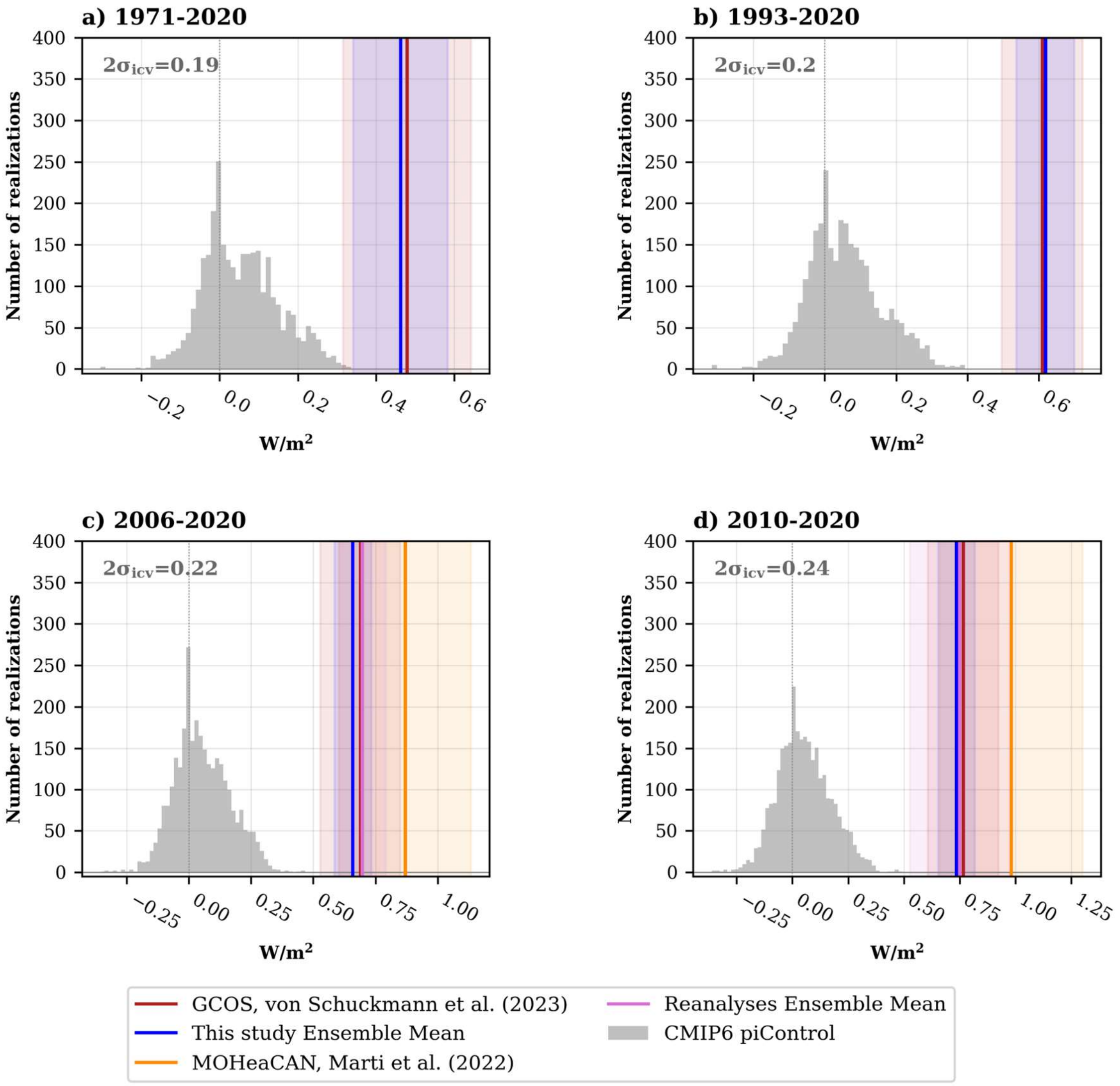

Figure S8 | Evaluation of GOHC trends from an ensemble of CMIP6 pre-industrial (piControl) simulations. Histogram of trends computed with a linear fit based on GOHC timeseries from CMIP6 piControl (3, 000 random samples from 46 model outputs, see Methods for details). GCOS (red), this study (blue), reanalysis ensemble (pink) and MOHeaCAN (orange) GOHC trends are also shown within their 95% confidence intervals (color shadings). The CMIP6 trends are calculated over various period lengths, corresponding to the length of the periods presented in Fig. 4, i.e **(a)** 50 years for 1971-2020, **(b)** 28 years for 1993-2020, **(c)** 15 years for 2006-2020. The 10-year period **(d)** is also added to assess the 10-year heating rates uncertainties in Fig. 5. In the upper left corner, the value of the two times standard deviation ($2\sigma_{icv}$) of the CMIP6 trends are displayed, with units expressed as W/m^2.

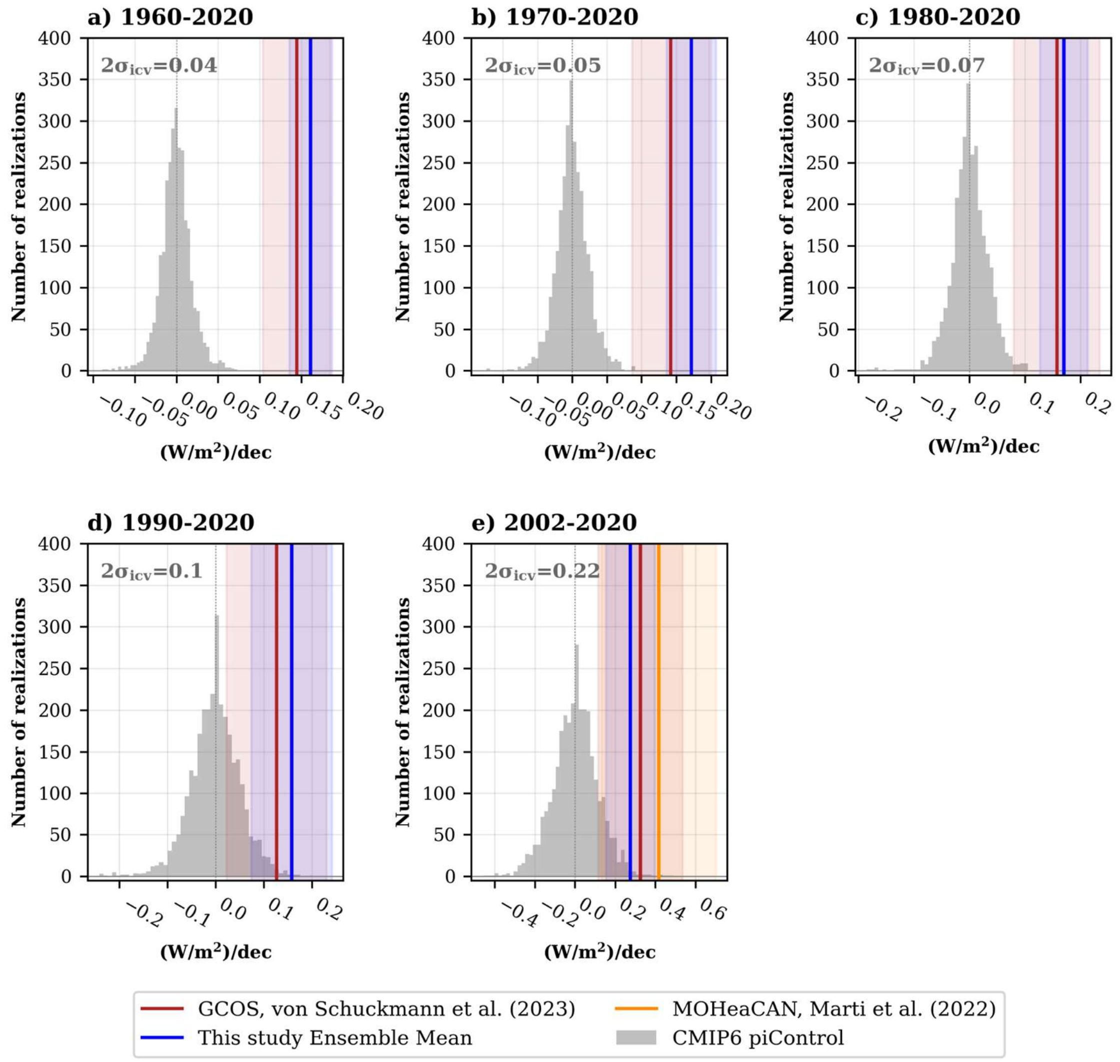

Figure S9 | Evaluation of GOHC accelerations from an ensemble of CMIP6 pre-industrial (piControl) simulations. Histogram of accelerations computed with a quadratic fit based on GOHC timeseries from CMIP6 piControl (3, 000 random samples from 46 model outputs, see Methods for details). GCOS (red), this study (blue) and MOHeaCAN (orange) GOHC accelerations are also shown within their 95% confidence intervals (color shadings). The CMIP6 accelerations are computed over various period lengths, corresponding to the length of the periods shown in Fig. 6, which include **(a)** 61 years for 1960-2020, **(b)** 51 years for 1970-2020, **(c)** 41 years for 1980-2020, **(d)** 31 years for 1990-2020 and **(e)** 19 years for 2002-2020. In the upper left corner, the value of the two times standard deviation ($2\sigma_{icv}$) of the CMIP6 accelerations are displayed, with units expressed as (W/m^2)/decade.

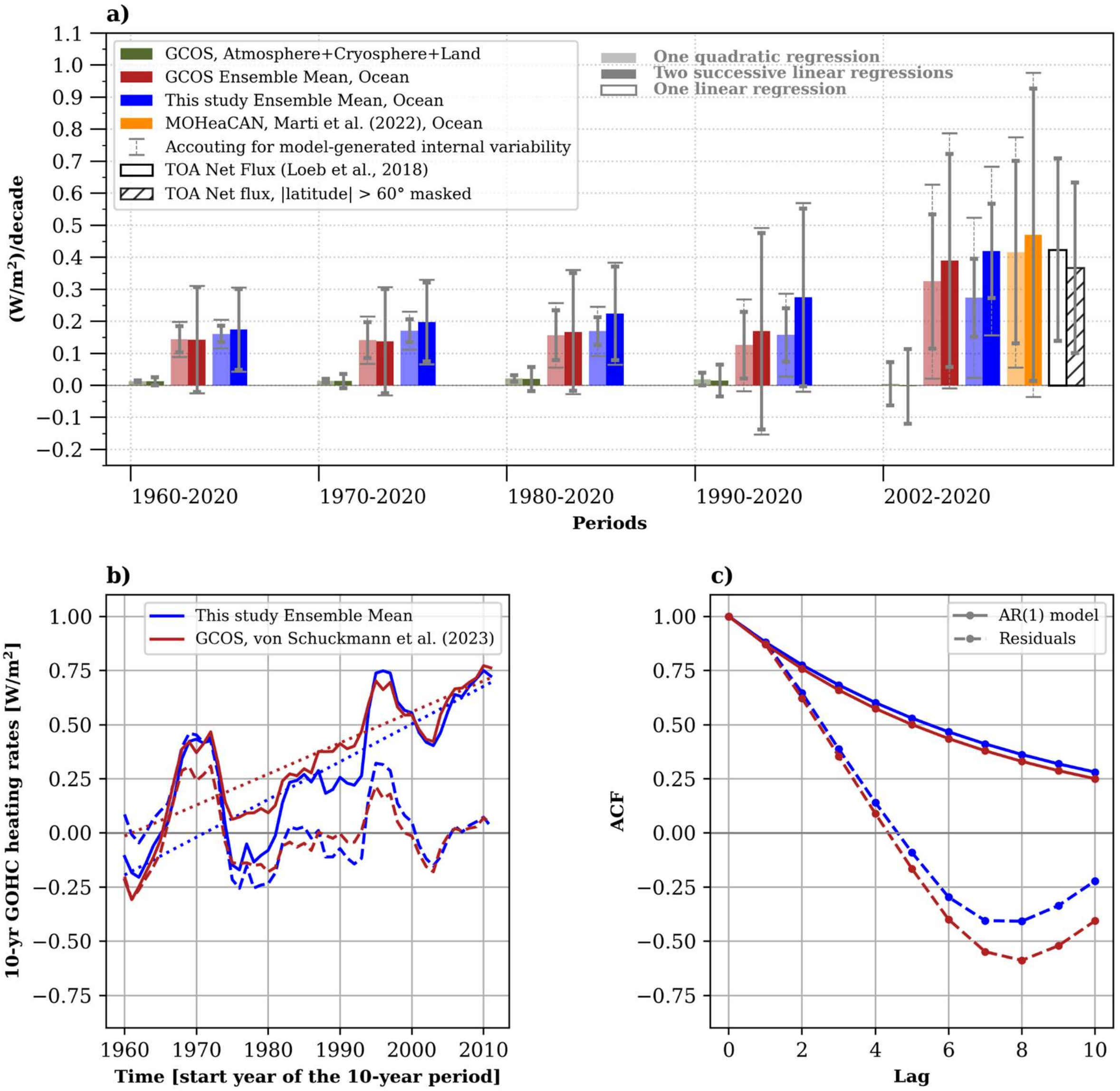

Figure S10 | Acceleration sensitivity to the method. (a) Earth system heating acceleration. The acceleration rates are estimated using a quadratic in time WLS regression of the annual GOHC time-series (light bars) (see Methods section) and a linear in time WLS regressions of the decadal heating rate time-series (dark bars). The in-situ ocean estimates from this study (blue bars) are compared to GCOS ocean estimates[9] (red bars), and satellite ocean estimates[19] (MOHeaCAN; orange bars). The non-ocean components (green bars) are estimated by summing the atmosphere, land and cryosphere GCOS[7] heat content time series. The top-of-atmosphere (TOA) estimates of warming acceleration (bars with black contours) are computed from a linear OLS regression accounting for autocorrelation[27], over global TOA net radiative flux (white bar) and near-global TOA net radiative flux (which excludes latitudes

higher than 60°, represented by the hatched white bar). Uncertainties for all estimates are shown at the 95% confidence level and take into account serial autocorrelation (solid gray lines) (see Methods). For the ocean heat content accelerations, the uncertainties taking into account model-generated internal variability are also displayed (dashed gray lines). A positive value indicates that the heat content is accelerating, while a negative value suggests deceleration. **(b)** 10-year linear trends computed from a WLS regression on GOHC from GCOS (solid red) and this study ensemble mean (solid blue), with associated linear fit (dotted lines) and fit residuals (dashed lines). **(c)** Autocorrelation function (ACF) of residuals computed for the first 10 lags over 10-year GOHC heating rates (dashed lines) and compared to the estimated AR(1) model (solid lines).

Supplementary Tables

Supplementary Table S1 | List of published Earth's Energy Imbalance absolute values or Global Ocean Heat Content trends synthesized in Fig. 1, with associated information and references.

Year start	Year end	EEI absolute value	EEI uncertainty	Informations	References
1955	2017	0,3	0,06	OHC (reconstruction, Green Function)	Zanna et al. (2019)[1]
1958	1973	0,06	0,08	OHC (in-situ, 0-2000m)	Cheng et al. (2022)[2]
1960	2015	0,4	0,09	EEI (in-situ inventory)	Cheng et al. (2017)[3]
1961	2010	0,33	0,25	EEI (TOA, AMIP)	Smith et al. (2015)[4]
1961	2010	0,36	0,15	EEI (TOA, CMIP5)	Smith et al (2015)[4]
1970	2014	0,45	0,1	OHC (reanalysis)	Dieng et al. (2017)[5]
1970	2010	0,45	0,08	OHC (in-situ 0-2000m)	Dieng et al. (2017)[5]
1971	2010	0,42	0,12	EEI (in-situ inventory)	AR5, IPCC 2013, Chapter 3 (ref. [6])
1971	2010	0,48	0,15	EEI (TOA, AMIP)	Smith et al. (2015)[4]
1971	2010	0,46	0,15	EEI (TOA, CMIP5)	Smith et al. (2015)[4]
1971	2018	0,47	0,1	EEI (in-situ inventory)	von Schuckmann et al. (2020)[7]
1971	2018	0,57	0,14	EEI (in-situ inventory)	AR6, IPCC 2021, Chapter 7 (ref. [8])
1971	2020	0,48	0,08	EEI (in-situ inventory)	von Schuckmann et al. (2023)[9]
1972	2005	0,48	0,32	EEI (CMIP5 inventory)	Cuesta-Valero et al. (2021)[10]
1975	2009	0,33	0,03	OHC (reanalysis)	Balmaseda et al. (2013)[11]
1991	2016	0,8	0,49	OHC (reconstruction based on atmospheric O2)	Resplandy et al. (2019)[12]
1993	2008	0,8	0,2	EEI (in-situ inventory)	Hansen et al. (2011)[13]
1993	2004	0,73	/	OHC (OPEN product, 0-2000m)	Su et al. (2021)[14]
1993	2010	0,56	0,13	EEI (in-situ inventory)	AR5, IPCC 2013, Chapter 3 (ref. [6])
1993	2010	0,59	0,27	EEI (TOA, AMIP)	Smith et al. (2015)[4]
1993	2010	0,68	0,14	EEI (TOA, CMIP5)	Smith et al. (2015)[4]
1993	2018	0,69	0,1	EEI (in-situ inventory)	von Schuckmann et al. (2020)[7]
1993	2018	0,72	0,17	EEI (in-situ inventory)	AR6, IPCC 2021, Chapter 7 (ref. [8])
1993	2020	0,68	0,11	EEI (in-situ inventory)	von Schuckmann et al. (2023)[9]
1993	2020	0,6	/	OHC (OPEN product, 0-2000m)	Su et al. (2021)[14]
1993	2021	0,62	0,18	OHC (OPEN product, 0-2000m)	Su et al. (2023)[15]
2005	2021	0,65	0,13	OHC (OPEN product, 0-2000m)	Su et al. (2023)[15]
2011	2021	0,78	0,21	OHC (OPEN product, 0-2000m)	Su et al. (2023)[15]
2000	2010	0,62	0,28	EEI (TOA, AMIP)	Smith et al. (2015)[4]
2000	2010	0,73	0,13	EEI (TOA, CMIP5)	Smith et al. (2015)[4]
2000	2016	0,62	0,1	EEI (TOA, reconstruction)	Liu et al. (2020)[16]
2000	2019	0,67	0,13	OHC (ARANN product)	Bagnell and DeVries (2021)[17]
2001	2009	0,84	0,1	OHC (reanalysis)	Balmaseda et al. (2013)[11]
2001	2010	0,5	0,43	EEI (TOA satellite)	Loeb et al. (2012)[18]
2002	2016	0,7	0,2	OHC (altim-GRACE)	Marti et al. (2022)[19]
2002	2016	0,57	0,18	OHC (altim-GRACE, Mascon only)	Marti et al. (2022)[19]
2003	2013	0,5	0,1	OHC (in-situ, 0-2000m)	Dieng et al. (2017)[5]
2003	2013	0,68	0,1	OHC (reanalysis)	Dieng et al. (2017)[5]
2003	2013	0,65	0,1	OHC (altim-GRACE)	Dieng et al. (2017)[5]
2003	2018	0,58	0,08	OHC (in-situ, 0-2000m)	Cheng et al. (2022)[2]
2003	2018	0,69	0,03	EEI (inventory)	Storto et al. (2022)[20]
2005	2010	0,58	0,15	EEI (in-situ inventory)	Hansen et al. (2011)[13]
2005	2014	0,9	0,3	EEI (in-situ inventory)	Trenberth et al. (2016)[21]
2005	2015	0,71	0,1	EEI (in-situ inventory)	Johnson et al. (2016)[22]
2005	2016	0,7	0,1	EEI (in-situ inventory)	von Schuckmann et al. (2018)[23]
2005	2015	0,59	0,13	OHC (in-situ, full-depth)	Marti et al. (2022)[19]
2005	2015	0,71	0,23	OHC (altim-GRACE)	Marti et al. (2022)[19]
2005	2015	0,56	0,21	OHC (altim-GRACE, Mascon only)	Marti et al. (2022)[19]
2005	2015	0,6	0,25	OHC (CMEMS)	Marti et al. (2022)[19]
2005	2018	0,8	0,1	EEI (in-situ inventory)	von Schuckmann et al. (2020)[7]
2005	2019	0,9	0,2	EEI (in-situ inventory)	Trenberth (2020)[24]
2005	2019	0,9	0,15	EEI (in-situ inventory)	Trenberth et al. (2022)[25]
2005	2019	0,76	0,17	EEI (in-situ inventory)	Hakuba et al. (2021)[26]
2005	2019	0,94	0,24	EEI (inventory, OHC altim-GRACE)	Hakuba et al. (2021)[26]
2005	2019	0,77	0,06	EEI (in-situ inventory)	Loeb et al. (2021)[27]
2005	2019	0,68	/	EEI (in-situ inventory)	Cheng et al. (2022)[2]
2006	2018	0,79	0,27	EEI (in-situ inventory)	AR6, IPCC 2021, Chapter 7 (ref. [8])
2006	2020	0,76	0,18	EEI (in-situ inventory)	von Schuckmann et al. (2023)[9]
2010	2018	0,87	0,12	EEI (in-situ inventory)	von Schuckmann et al. (2020)[7]

Supplementary Table S2 | Data used in this study to estimates GOHC time series, trends and accelerations, together with informations related on data pre-processing, spatio-temporal coverage and references.

Data name	Data Type	Informations	Time coverage	Grid spacing	Reference and download link
IAP	3D temperature dataset and GOHC timeseries **Argo+others**	**Mapping method**: Ensemble Optimal Interpolation (EnOI) method **First guess**: use of 40 CMIP5 models **XBT correction**: Cheng et al. (2014)[28] **MBT correction**: Gouretski and Cheng (2020)[29] **Uncertainty estimate on OHC**: EnOI framework used to propagate instrumental error and mapping uncertainty into final estimate. Global error is computed by ensemble members.	1960-2020 (monthly)	1°x1° (Global)	Cheng et al. (2017)[3] ftp://www.ocean.iap.ac.cn/cheng/CZ16_v3_IAP_Temperature_gridded_1month_netcdf/
EN4.4.2.2	3D temperature dataset **Argo+others**	**Mapping method**: Objective Interpolation (OI) **First guess:** 1970-2000 climatology **XBT correction**: 4 options including Cheng et al. (2014)[28]; Levitus et al. (2009)[30]; Gouretski and Reseghetti (2010)[31]; Cowley et al. (2013)[32] **MBT correction**: 3 options including Gouretski and Cheng (2020)[29]; Gouretski and Reseghetti (2010)[31]; Levitus et al. (2009)[30] **Uncertainty estimate on OHC**: not provided	1960-2020 (monthly)	1°x1° (Global)	Good et al. (2013)[33] https://hadleyserver.metoffice.gov.uk/en4/download.html
NOAA	3D temperature dataset **Argo+others**	**Mapping method**: OI **First guess**: climatology (zero anomaly) **Uncertainty estimate on OHC**: not provided	2005-2020 (monthly)	1°x1° (Global)	Levitus et al. (2012)[34] https://www.ncei.noaa.gov/access/global-ocean-heat-content/bin/anomalydata_tm.pl#ShowNote
JAMSTEC	3D temperature dataset **Argo+others**	**Mapping method**: OI **First guess:** WOA01 seasonal climatology **Uncertainty estimate on OHC**: not provided	2005-2020 (monthly)	1°x1° (Global)	Hosoda et al. (2008)[35] http://www.jamstec.go.jp/ARGO/argo_web/argo/?page_id=83&lang=en
ISAS	3D temperature dataset **Argo only**	**Mapping method**: OI **First guess:** WOA18 2005-2017 monthly climatology **Uncertainty estimate on OHC**: not provided	2005-2020 (monthly)	0.5°x0.5° (Global)	Kolodziejczyk et al. (2021)[36] https://doi.org/10.17882/52367
GDCSM	3D temperature dataset **Argo only**	**Mapping method**: Gradient-Dependent OI **First guess:** climatology (zero anomaly) **Uncertainty estimate on OHC**: not provided	2005-2020 (monthly)	0.5°x0.5° (Global)	Zhang et al. (2022)[37] http://data.argo.org.cn/pub/ARGO/GDCSM/
CSIO/BOA-Argo	3D temperature dataset **Argo only**	**Mapping method**: OI (Barnes, 1973)[38] **First guess:** Cressman successive correction method (Cressman, 1959)[39] used on argo data. **Uncertainty estimate on OHC**: not provided	2005-2020 (monthly)	1°x1° (79.5°S-79.5°N)	Li et al. (2017)[40] ftp://data.argo.org.cn/pub/ARGO/BOA_Argo/
SCRIPPS	3D temperature dataset **Argo only**	**Mapping method**: OI (Bretherton et al., 1976)[41] **First guess:** A weighted least-squares fit to nearby data was used each month **Uncertainty estimate on OHC**: not provided	2005-2020 (monthly)	1°x1° (70°S-67°N)	Roemmich and Gilson (2009)[42] http://sio-argo.ucsd.edu/RG_Climatology.html
IPRC	3D temperature dataset **Argo only**	**Mapping method**: Variational analysis **First guess:** not mentioned **Uncertainty estimate on OHC**: not provided	2005-2020 (monthly)	1°x1° (Global)	http://apdrc.soest.hawaii.edu/projects/Argo/data/gridded/On_standard_levels/index-1.html

von Schuckmann and Le Traon, 2011	GOHC time series **Argo only**	**Mapping method**: Weighted averaging method (Bretherton et al., 1976)[41] using non-gridded Argo only temperature profiles<br>**First guess:** global mean of observations<br>**Uncertainty estimate on OHC**: uncertainty accounting from instrumental error , mapping uncertainty, and the choice of the climatology	2005-2020 (annual)	No gridded field available<br><br>60°N-60°S, shallow area (bathymetry <300m) excluded	von Schuckmann and Le Traon (2011)[43]<br><br>Copernicus Marine, 2022: Global Ocean Heat Content (0-2000m) time series and trend from Reanalysis & Multi-Observations Reprocessing. Copernicus Marine Ocean Monitoring Indicator, doi: 10.48670/moi-00235, https://data.marine.copernicus.eu/product/GLOBAL_OMI_OHC_area_averaged_anomalies_0_2000.
GCOS	GOHC time series	**OHC estimation**: Ensemble mean of 16 in-situ GOHC time series<br>**Uncertainty estimate on OHC :** 2*Ensemble spread	1960-2020 (annual)	No gridded field available<br><br>60°N-60°S, shallow area (bathymetry <300m) excluded	von Schuckmann et al. (2023)[9]<br>https://www.wdc-climate.de/ui/entry?acronym=GCOS_EHI_1960-2020
Reanalyses	GOHC time series	**OHC estimation**: Ensemble mean of 3 GOHC time series estimated from the reanalyses GLORYS, CGLOR, OSRS5<br><br>**Uncertainty estimate on OHC :** 2*Ensemble spread	2005-2020 (annual)	No gridded field available<br><br>60°N-60°S, shallow area (bathymetry <300m) excluded	Copernicus Marine, 2022: Global Ocean Heat Content (0-2000m) time series and trend from Reanalysis & Multi-Observations Reprocessing. Copernicus Marine Ocean Monitoring Indicator, doi: 10.48670/moi-00235, https://data.marine.copernicus.eu/product/GLOBAL_OMI_OHC_area_averaged_anomalies_0_2000.
MOHeaCAN	GOHC time series	**OHC estimation**: Thermosteric sea level is estimated from the total seal level (altimetry) minus its ocean mass component (GRACE), neglecting halosteric effect, and is then converted into ocean heat content using a coefficient of expansion efficiency of heat estimated from in-situ temperature data.<br>**Uncertainty estimate on OHC :** provided following the method of Ablain et al. (2019)[45], i.e. uncertainties are propagated from altimetry and ocean mass data to GOHC estimate.	2002-2020 (monthly)	1°×1° (66°N-66°S)	Marti et al. (2022)[19]<br>https://www.aviso.altimetry.fr/en/data/products/ocean-indicators-products/ocean-heat-content-and-earth-energy-imbalance/global-ocean-heat-content-change-and-earth-energy-imbalance.html

Supplementary Discussion

Analysis of trends, acceleration and associated uncertainties estimates

1. Comparison of structural and internal uncertainties

In Fig. 2, we compared GCOS and this study GOHC structural uncertainty to the internal uncertainty of the historic IAP product[3], in the estimate of this study in-situ GOHC timeseries and trends. We extend this analysis to two other historic products which also provide their own internal uncertainty, i.e. the Ishii et al. (2017)[44] and Levitus et al. (2012)[34] GOHC timeseries (Fig. S3a). We use the WLS regression to compute GOHC trends (Fig. S3b) and accelerations (Fig. S3c) (see Methods). Except for the more recent period, where the internal uncertainty of the Ishii et al. (2017) product is larger or of the same order of magnitude (in yellow in Fig. S3), the structural uncertainties (this study and GCOS) encompass the internal uncertainties of all three historical products. This is true for GOHC (Fig. S3a), its trend (Fig. S3b) and its acceleration (Fig. S3c). The central estimates of GOHC trends are not impacted by the uncertainty used in the weighting matrix of the WLS regression (Fig. S3b). In the same way, the 1960-2020, 1970-2020, 1980-2020, 1990-2020 acceleration values are weakly impacted by the type of uncertainty used in the WLS regression, i.e., by less than 0.1 W/m^2/decade (Fig. S3c). However, we note that over 2002-2020, acceleration estimates are more sensitive and can exceed 0.1 W/m^2/decade depending on the uncertainty type used in the weighting matrix of the WLS regression (Fig. S3c). We note in particular that the use of Ishii or Levitus internal uncertainties in the weighting matrix does not support an increased acceleration rate over 2002-2020 as compared to the longer-term period. The increase in acceleration rate over 2002-2020 is, however, clearly visible in the case of the two successive WLS regressions, regardless of the type of uncertainties used in the weighting matrix (Fig. S3c).

2. Comparison of regression methods for estimating trends

We also investigated the impact of the method used to calculate GOHC trends (Fig. S4a and S5a), by testing the WLS, OLS, LOWESS, and QUADRATIC regressions (see Methods). Results are shown for this study's GOHC in Fig. S4a and for GCOS in Fig. S5a. The decadal trend uncertainties given by the WLS regression largely encompasses the uncertainties given by the other methods (Fig. S4a and S5a, dark blue envelope). The central estimates of the 10-year WLS and OLS trends are nearly the same, and displayed substantial variability, superimposed to a long-term upward trend in accordance with the evolution of the 10-year heating rates calculated using the 'Delta' method based on the quadratic (yellow curve) and LOWESS (red curve) GOHC fits (Fig. S4a and S5a).

3. Comparison of two different approaches for estimating accelerations

In Fig. S4, S5, S6, and S10, we explore two distinct methods for assessing heat content acceleration. The first method, employed in the article, involves determining acceleration values by fitting a quadratic curve to the heat content time series (illustrated in Fig. S4b, S5b, S6b, and depicted as light bars in Fig. S10). The second approach consists in calculating linear trends based on 10-year heating rates curves (from Fig. 5) over various time spans presented in Fig. 6 (i.e., 1960-2020, 1970-2020, 1980-2020, 1990-2020, and 2002-2020). The results for this second approach are shown in Fig. S4c, S5c, S6a, and represented by dark bars in Fig. S10.

In Fig. S4b, we show that the accelerations computed via OLS and WLS quadratic regressions over the GOHC time series are nearly identical, and the uncertainty provided by the WLS method either encompasses or is comparable to the uncertainties presented by the OLS regression. These findings remain consistent when the analysis is applied to GOHC time series from GCOS, as shown in Fig. S5b.

In Fig. S4c and Fig. S5c, we show that the acceleration values obtained through a linear fit on 10-year heating rates (derived from LOWESS, QUADRATIC, OLS, and WLS methods over the GOHC time series, refer to the Methods section for details) are quite similar for periods starting before 2002. However, it is important to note that acceleration values for the 2002-2020 period are highly dependent

on the chosen approach, with differences exceeding 0.30 (W/m^2)/decade between accelerations computed using the LOWESS and OLS methods (as depicted in Fig. S4c and Fig. S5c).

Nevertheless, the linear fit applied to 10-year heating rates introduces notable uncertainties in acceleration, as demonstrated in Fig. S4c, S5c, S6a, and S10. This increased uncertainty results from the intentionally induced high autocorrelation in the 10-year heating rates time series, which stems from the computation of heating rates across overlapping windows (refer to Fig. S10b and c). In addition, we provide the autocorrelation function for the linear fit applied to the 10-year heating rates in Fig. S10c, illustrating that the autocorrelation structure of the residuals in this method differs from an AR(1) model, in contrast to the quadratic approach (as depicted in Fig. S7b and d). Consequently, in the main text, we base our assessment of acceleration and associated uncertainties solely on the quadratic fit approach.

4. Acceleration sensitivity to the product used.

In this section, we investigate the acceleration of the GOHC for each product used in our GOHC ensemble mean, to understand if the GOHC accelerations shown in Fig. 6 (blue bars) are an artefact of the construction of our GOHC ensemble mean, or whether these accelerations are captured in each of the products of the ensemble. For each of the products used in our ensemble, we compare the acceleration values given by the WLS linear regressions performed on 10-year heating rates (Fig. S6a) with those from the WLS quadratic regression (Fig. S6b). We also compute the acceleration over the period 2005-2020 to benefit from a wider range of products.

Regardless of the method, almost all products show an acceleration significantly different from zero over the periods 1960-2020, 1970-2020, 1980-2020. Over 1960-2020, 1970-2020 and 1980-2020, the acceleration values from one product to another are similar and remain quite constant (for both approaches) and are consistent with the GOHC ensemble mean acceleration rate. However, the acceleration values differ from one product to another over the period 1990-2020, 2002-2020 and 2005-2020. Over 2002-2020, all products show an increase relative to longer periods in acceleration rates for both approaches. Over 2005-2020, except for IAP, JAMSTEC, IPRC and von Schuckmann and Le

Traon (2011) products which show non-significant GOHC acceleration rates, all the others products show significant GOHC acceleration rates ranging from 0.26±0.13 (W/m^2)/decade to 0.50±0.13 (W/m^2)/decade, which are higher values than the 1960-2020 acceleration, ranging from 0.14±0.08 (W/m^2)/decade to 0.20±0.13 (W/m^2)/decade (for both methods taken together).

References for Supplementary Informations

1. Zanna, L., Khatiwala, S., Gregory, J. M., Ison, J. & Heimbach, P. Global reconstruction of historical ocean heat storage and transport. *Proc. Natl. Acad. Sci.* **116**, 1126–1131 (2019).
2. Cheng, L., Foster, G., Hausfather, Z., Trenberth, K. E. & Abraham, J. Improved Quantification of the Rate of Ocean Warming. *J. Clim.* **35**, 4827–4840 (2022).
3. Cheng, L. *et al.* Improved estimates of ocean heat content from 1960 to 2015. *Sci. Adv.* **3**, e1601545 (2017).
4. Smith, D. M. *et al.* Earth's energy imbalance since 1960 in observations and CMIP5 models. *Geophys. Res. Lett.* **42**, 1205–1213 (2015).
5. Dieng, H. B., Cazenave, A., Meyssignac, B., von Schuckmann, K. & Palanisamy, H. Sea and land surface temperatures, ocean heat content, Earth's energy imbalance and net radiative forcing over the recent years. *Int. J. Climatol.* **37**, 218–229 (2017).
6. *Climate change 2013: The Physical Basis. Contribution of Working Group I to the Fifht Assessment Report of the Intergovernmental Panel on Climate Change*. (2013).
7. von Schuckmann, K. *et al.* Heat stored in the Earth system: where does the energy go? *Earth Syst. Sci. Data* **12**, 2013–2041 (2020).
8. Forster, P. *et al.* The Earth's energy budget, climate feedbacks, and climate sensitivity. in *Climate Change 2021: The Physical Science Basis. Contribution of Working Group I to the Sixth Assessment Report of the Intergovernmental Panel on Climate Change* (eds. Masson-Delmotte, V. et al.) 923–1054 (Cambridge University Press, 2021). doi:10.1017/9781009157896.001.
9. von Schuckmann, K. *et al.* Heat stored in the Earth system 1960–2020: where does the energy go? *Earth Syst. Sci. Data* **15**, 1675–1709 (2023).
10. Cuesta-Valero, F. J., García-García, A., Beltrami, H. & Finnis, J. First assessment of the earth heat inventory within CMIP5 historical simulations. *Earth Syst. Dyn.* **12**, 581–600 (2021).
11. Balmaseda, M. A., Trenberth, K. E. & Källén, E. Distinctive climate signals in reanalysis of global ocean heat content. *Geophys. Res. Lett.* **40**, 1754–1759 (2013).
12. Resplandy, L. *et al.* Quantification of ocean heat uptake from changes in atmospheric O2 and

CO_2 composition. *Sci. Rep.* **9**, 20244 (2019).

13. Hansen, J., Sato, M., Kharecha, P. & von Schuckmann, K. Earth's energy imbalance and implications. *Atmospheric Chem. Phys.* **11**, 13421–13449 (2011).

14. Su, H., Qin, T., Wang, A. & Lu, W. Reconstructing Ocean Heat Content for Revisiting Global Ocean Warming from Remote Sensing Perspectives. *Remote Sens.* **13**, 3799 (2021).

15. Su, H., Wei, Y., Lu, W., Yan, X.-H. & Zhang, H. Unabated Global Ocean Warming Revealed by Ocean Heat Content from Remote Sensing Reconstruction. *Remote Sens.* **15**, 566 (2023).

16. Liu, C. *et al.* Variability in the global energy budget and transports 1985–2017. *Clim. Dyn.* **55**, 3381–3396 (2020).

17. Bagnell, A. & DeVries, T. 20th century cooling of the deep ocean contributed to delayed acceleration of Earth's energy imbalance. *Nat. Commun.* **12**, 4604 (2021).

18. Loeb, N. G. *et al.* Observed changes in top-of-the-atmosphere radiation and upper-ocean heating consistent within uncertainty. *Nat. Geosci.* **5**, 110–113 (2012).

19. Marti, F. *et al.* Monitoring the ocean heat content change and the Earth energy imbalance from space altimetry and space gravimetry. *Earth Syst. Sci. Data* **14**, 229–249 (2022).

20. Storto, A., Cheng, L. & Yang, C. Revisiting the 2003–18 Deep Ocean Warming through Multiplatform Analysis of the Global Energy Budget. *J. Clim.* **35**, 4701–4717 (2022).

21. Trenberth, K. E., Fasullo, J. T., Schuckmann, K. von & Cheng, L. Insights into Earth's Energy Imbalance from Multiple Sources. *J. Clim.* **29**, 7495–7505 (2016).

22. Johnson, G. C., Lyman, J. M. & Loeb, N. G. Improving estimates of Earth's energy imbalance. *Nat. Clim. Change* **6**, 639–640 (2016).

23. von Schuckmann, K. *et al.* Copernicus Marine Service Ocean State Report. *J. Oper. Oceanogr.* **11**, S1–S142 (2018).

24. Trenberth, K. E. Understanding climate change through Earth's energy flows. *J. R. Soc. N. Z.* **50**, 331–347 (2020).

25. Trenberth, K. E. & Cheng, L. A perspective on climate change from Earth's energy imbalance. *Environ. Res. Clim.* **1**, 013001 (2022).

26. Hakuba, M. Z., Frederikse, T. & Landerer, F. W. Earth's Energy Imbalance From the Ocean

Perspective (2005–2019). *Geophys. Res. Lett.* **48**, e2021GL093624 (2021).

27. Loeb, N. G. *et al.* Satellite and Ocean Data Reveal Marked Increase in Earth's Heating Rate. *Geophys. Res. Lett.* **48**, e2021GL093047 (2021).

28. Cheng, L., Zhu, J., Cowley, R., Boyer, T. & Wijffels, S. Time, Probe Type, and Temperature Variable Bias Corrections to Historical Expendable Bathythermograph Observations. *J. Atmospheric Ocean. Technol.* **31**, 1793–1825 (2014).

29. Gouretski, V. & Cheng, L. Correction for Systematic Errors in the Global Dataset of Temperature Profiles from Mechanical Bathythermographs. *J. Atmospheric Ocean. Technol.* **37**, 841–855 (2020).

30. Levitus, S. *et al.* Global ocean heat content 1955–2008 in light of recently revealed instrumentation problems. *Geophys. Res. Lett.* **36**, (2009).

31. Gouretski, V. & Reseghetti, F. On depth and temperature biases in bathythermograph data: Development of a new correction scheme based on analysis of a global ocean database. *Deep Sea Res. Part Oceanogr. Res. Pap.* **57**, 812–833 (2010).

32. Cowley, R., Wijffels, S., Cheng, L., Boyer, T. & Kizu, S. Biases in Expendable Bathythermograph Data: A New View Based on Historical Side-by-Side Comparisons. *J. Atmospheric Ocean. Technol.* **30**, 1195–1225 (2013).

33. Good, S. A., Martin, M. J. & Rayner, N. A. EN4: Quality controlled ocean temperature and salinity profiles and monthly objective analyses with uncertainty estimates. *J. Geophys. Res. Oceans* **118**, 6704–6716 (2013).

34. Levitus, S. *et al.* World ocean heat content and thermosteric sea level change (0–2000 m), 1955–2010. *Geophys. Res. Lett.* **39**, (2012).

35. Hosoda, S., Ohira, T. & Nakamura, T. A monthly mean dataset of global oceanic temperature and salinity derived from Argo float observations. *JAMSTEC Rep. Res. Dev.* **8**, 47–59 (2008).

36. Kolodziejczyk, N., Prigent-Mazella, A. & Gaillard, F. ISAS temperature and salinity gridded fields. (2021) doi:10.17882/52367.

37. Zhang, C. *et al.* Global Gridded Argo Dataset Based on Gradient-Dependent Optimal Interpolation. *J. Mar. Sci. Eng.* **10**, 650 (2022).

38. Barnes. *Mesoscale Objective Map Analysis Using Weighted Time-series Observations*. (1973).

39. Cressman, G. P. AN OPERATIONAL OBJECTIVE ANALYSIS SYSTEM. *Mon. Weather Rev.* **87**, 367–374 (1959).

40. Li, H. *et al.* Development of a global gridded Argo data set with Barnes successive corrections. *J. Geophys. Res. Oceans* **122**, 866–889 (2017).

41. Bretherton, F. P., Davis, R. E. & Fandry, C. B. A technique for objective analysis and design of oceanographic experiments applied to MODE-73. *Deep Sea Res. Oceanogr. Abstr.* **23**, 559–582 (1976).

42. Roemmich, D. & Gilson, J. The 2004–2008 mean and annual cycle of temperature, salinity, and steric height in the global ocean from the Argo Program. *Prog. Oceanogr.* **82**, 81–100 (2009).

43. von Schuckmann, K. & Le Traon, P.-Y. How well can we derive Global Ocean Indicators from Argo data? *Ocean Sci.* **7**, 783–791 (2011).

44. Ishii, M. *et al.* Accuracy of Global Upper Ocean Heat Content Estimation Expected from Present Observational Data Sets. *Sola* **13**, 163–167 (2017).

45. Ablain, M. *et al.* Uncertainty in satellite estimates of global mean sea-level changes, trend and acceleration. *Earth Syst. Sci. Data* **11**, 1189–1202 (2019).
